# Processing references in context: when *the* polar bear does not meet *a* polar bear

**DOI:** 10.1007/s10339-023-01150-4

**Published:** 2023-07-15

**Authors:** Bettina Rolke, Mareike Kirsten, Verena C. Seibold, Susanne Dietrich, Ingo Hertrich

**Affiliations:** 1 Evolutionary Cognition, Department of Psychology, University of Tuebingen, Schleichstraße 4, 72076 Tuebingen, Germany; 2 Hertie Institute for Clinical Brain Research, Tuebingen, Germany

**Keywords:** Discourse understanding, Context reference process, Presupposition, Cognitive processing, Semantics, Definite and indefinite determiner

## Abstract

Discourse understanding is hampered when missing or conflicting context information is given. In four experiments, we investigated what happens (a) when the definite determiner “the,” which presupposes existence and uniqueness, does not find a unique referent in the context or (b) when the appropriate use of the indefinite determiner is violated by the presence of a unique referent (Experiment 1 and Experiment 2). To focus on the time-course of processing the uniqueness presupposition of the definite determiner, we embedded the determiner in different sentence structures and varied the context (Experiment 3 and Experiment 4). Reading time served as an index of processing difficulty in a word-by-word self-paced reading task and acceptability judgments provided hints for a possible repair of a presupposition violation. Our results showed that conflicting and missing context information lowered acceptability ratings and was associated with prolonged reading times. The pattern of results differed depending on the nature of the presupposition (Experiments 1 and 2) and whether supplementing missing context information was possible (Experiment 3 and Experiment 4). Our findings suggest that different cognitive processes come into play when interpreting presuppositions in order to get a meaningful interpretation of a discourse.

## Introduction

To understand a discourse, it is necessary to keep track of discourse entities as well as context information. While reading or listening, relevant information is gathered and stored in memory so that it can be recalled and connected to future incoming information. An inherent assumption is that motivated recipients aim to accumulate information during the course of a conversation or a reading process. Thus, they use inferential processes to establish a coherent and meaningful content of the current discourse (Kintsch and van Dijk 1978). Luckily, in language processing, certain aids can help glue pieces of information from different discourse constituents together. One class of this “linguistic glue” is the use of presuppositions. A presupposition is generally defined as meaning that is assumed to be backgrounded or part of the discourse context rather than asserted by a sentence (e.g., Krahmer 1998; Schwarz 2009, 2016).

Typically, presuppositions are triggered by specific lexical items, so-called presupposition triggers. The present study focusses on cognitive processing of two presupposition triggers, the German definite determiners (“*der,*” “*die,*” “*das*”; English “*the*”) and the indefinite determiners (“*ein,*” “*eine*”; English “*a*”). Different functions of the two determiners have been proposed. According to Strawson (1950), the definite determiner presupposes the existence of a unique entity. For example, the sentence “*The king of France is bald*” will have a referent if there is a king of France. Krahmer (1998, see also Lewis 1979), however, argued that this mere *existence* presupposition is not enough to describe the meaning of the definite determiner; one important feature of the definite determiner is its reference to the most salient or even to a unique discourse entity (i.e., the definite determiner presupposes *uniqueness* of a referent). Several other interpretations of the meaning of the definite determiner have been proposed (for an overview, see, e.g., Krahmer 1998), and the issue of which specific presuppositions the definite determiner carries is still under debate. However, it seems fair to say that the common assumption of most theories is that the definite determiner triggers the presupposition that there exists a discourse entity to which a reference is needed. Most theories also seem to agree that a sentence is left infelicitous when this reference process fails to find a discourse referent (but see Russell 1905).

Whereas the appropriate use of the definite determiner requires a given and unique discourse referent, the indefinite determiner is assumed to need either zero or more than one discourse referent(s). The latter case, the *anti-uniqueness* presupposition caused by the indefinite determiner is, for example, suggested by Alonso-Ovalle et al. (2009; see also Hawkins 1991; Heim 1991). Regarding the former case (absence of a referent), several authors assign a *novelty* presupposition to the indefinite determiner (Anderson and Holcomb 2005; Heim 1982; Krahmer 1998). This novelty presupposition holds that the indefinite determiner introduces a new discourse entity, which has to be unfamiliar within the context to avoid a failure of presupposition processing. Thus, when a unique discourse entity is familiar, the definite determiner should be used. Taken together, linguistic theories propose different roles for the definite and the indefinite determiner. These roles are mainly defined by the availability and the number of introduced discourse entities.

The theoretical assumptions concerning the role of the definite and the indefinite determiner as presupposition triggers outlined so far raise the question of how sentences containing these triggers are cognitively processed. From a theoretical perspective, it seems plausible to assume that processing of the definite determiner differs from processing of the indefinite determiner: The definite determiner triggers a reference process (e.g., Altmann 1998) which aims to bind an anaphoric noun phrase to its discourse antecedent. The indefinite determiner, however, does not require a reference process to a discourse antecedent, but it triggers a process to check whether the anti-uniqueness condition is given or whether a new item has to be introduced in the discourse. According to the Givenness hierarchy proposed by Gundel et al. (1993; see also Gundel et al. 2012), the definite determiner has a higher hierarchical status than the indefinite one as it restricts the set of potential referents in referring to a uniquely identifiable entity. This is in contrast to the indefinite determiner, which only allows identification of a type of described thing. Gundel et al.’s framework includes the speaker’s intention and, in this sense, the chosen determiners serve as signals to the addressee concerning the availability of a referent in the addressee's memory. The definite determiner signals that a unique referent should exist in long-term or short-term memory, and the indefinite determiner signals that a salient and unique referent is unlikely to be found in memory. The authors argue that the type-identifiable interpretation of the indefinite determiner should require less processing capacity because it is easier to access than the more referential one of the definite determiner.

Apart from these theoretical considerations, empirical studies on processing of the definite and indefinite determiners have already shed some light into the cognitive processing of presuppositions and the definite and indefinite determiner in more particular. The basic approach herein is to create a situation in which a presupposition trigger is used in a sentence, but the presupposition is not supported by the context (e.g., Altman and Steedman 1988; Domaneschi and die Paola 2018; Garnahm et al. 1997; Haviland and Clark 1974; Schneider 2020). According to linguistic theories, this situation should lead to a presupposition failure. This failure should make the sentence uninterpretable (cf. Heim and Kratzer 1998, for example) or undefined (Strawson 1950) unless the discourse recipient saves the meaning of the sentence by adding missing information to the discourse (Beaver and Zeevat 2007; Kadmon 2001; Lewis 1979; Stalnaker 1973)—a process that has been referred to as accommodation. Importantly, from a cognitive perspective, processing violated assumptions triggered by the definite and by the indefinite determiner as well as the attempt to accommodate should require (additional) cognitive processing resources.

Previous studies have already thematized the question of how unfulfilled presuppositions are processed. In an early study, Haviland and Clark (1974) investigated presuppositions in the context of the definite determiner by measuring comprehension time for sentences. The authors showed that the comprehension time for a test sentence was increased when the presupposed information was not given in the preceding context sentence. This result suggests that some additional cognitive processing is required in the latter case, due to increased cognitive load. Altmann and Steedmann (1988; see also Garnham et al. 1997) addressed the question of which specific time point the presupposition is processed within a sentence. The authors employed a self-paced phrase-by-phrase reading task in order to track the time-course of definite determiner processing. The authors induced presupposition failures by creating context sentences that included two reference candidates (two safes) for the definite determiner in a test sentence, for example “*A burglar broke into a bank carrying some dynamite.... Once inside he saw that there was a safe which had a new lock and a safe which had an old lock*.”. Subsequently to the presentation of the context sentence, they presented test sentences like “*The burglar/ blew open/ the safe/ with the new lock/ and made off/ with the loot*,” or “*The burglar/ blew open/ the safe/ with the dynamite/ and made off/ with the loot.*” In contrast to the first test sentence, which allows the usage of the definite noun phrase (NP) because the (underlined) restrictive insertion guarantees uniqueness of the safe in the context, the second test sentence could not be saved by the insertion. The results revealed shorter reading times for the (underlined) disambiguating phrase when the context supported the presupposition of the test sentence. This result has been interpreted by the authors as an immediate increase of cognitive processing load at the moment the processor notices that the context does not support a presupposition (Altmann and Steedmann 1988). Moreover, Domaneschi and Di Paola (2018; see also Schneider et al. 2020) have observed more specific evidence on the time-course of presupposition processing by comparing different types of presupposition triggers (i.e., the definite determiner “*the*”, change of state verbs such as “*to give up*”, iterative expressions such as “*again*”, and focal particles such “*also/too*”) in a word-by-word reading time study. They reported longer reading times when a neutral context did not mention a suitable referent for the triggered presupposition compared to when it was explicitly mentioned in the context sentence. Most importantly, the first effect on reading times in a sentence containing an unfulfilled presupposition occurred at the word following the trigger (*T* + 1), that is, shortly after participants had parsed the presupposition trigger. Taken together, the results of these reading time studies suggest that unfulfilled presuppositions are reflected in longer reading times and that presupposition processing starts at a very early time point in sentence comprehension.

A few studies have directly compared the processing of the definite and the indefinite determiner, but these studies have provided somewhat mixed results: Whereas some studies (Murphy 1984; Gernsbacher and Robertson 2002) have revealed evidence for higher processing demands when using the indefinite in comparison to the definite, others obtained evidence for the opposite relationship (Schumacher 2009). For instance, Murphy (1984) presented sentences like “*Though driving 55, Steve was passed by a truck. Later, George was passed by a/the truck too*.” He obtained longer comprehension times for sentences containing the indefinite than those containing the definite (see also Gernsbacher and Robertson 2002). This result was taken as evidence that the introduction of a new discourse entity in case of the indefinite determiner requires extra cognitive costs compared to referring to an existing referent with the definite determiner (see also Clifton 2013) or “mapping” existing entities into a “*structure building framework*” (e.g., Gernsbacher and Robertson 2002). One the other hand, however, the results of some electrophysiological studies speak in favor of higher processing demands for the definite compared to the indefinite determiner (Schumacher 2009; Anderson and Holcomb 2005). Specifically, Schumacher (2009) compared ERPs evoked by reading German sentences containing a definite or an indefinite determiner. She observed that the definite determiner was associated with an enhanced frontal ERP (the left anterior negativity [LAN]), relative to the indefinite determiner. To explain this result, Schumacher (2009) suggested that the identification of a unique referential entity within working memory that is associated with the definite determiner is cognitively more demanding than the introduction of a new entity associated with the indefinite determiner. These results seem to be more compatible with the hierarchical Givenness assumptions offered by Gundel et al. (1993, 2012). Finally, in a phrase-by-phrase-reading time study Clifton (2013) compared the processing of the definite and the indefinite determiner by presenting a noun phrase (NP; “*the stove*/*a stove*”) in context situations familiar to the readers, who were expected to assume that some contexts contained one single referent (e.g., “*In the kitchen*…”) while others contained more than one referent (e.g., “*In the appliance store*…”). In contrast to the above-described ERP studies, the author did not find a general difference in reading times between determiner types, but instead found increased reading times for both determiner types when participants read the less compatible phrase-NP combination (e.g., “In the kitchen” with “a stove”). This “unfulfillment” effect, however, occurred only when participants had to work on an arithmetic task alongside the main task to increase working memory load. The author concluded that participants have to be forced to process the sentences at a deep semantical level to obtain (mis)fulfilled effects for presupposition processing. Hence, although this study shows that the inappropriate use of the indefinite determiner prolongs reading times (as has been previously shown for the definite determiner), the raw effect of unfulfillment in this study prevents any conclusion concerning differential cognitive processes associated with the two determiners.

The question of how different presupposition triggers are cognitively processed becomes more difficult if one takes into account that a trigger can induce different processing requirements in different contexts. By using electrophysiological methods, Burkhardt (2008; see also Schwarz 2009) investigated whether inherently definite noun phrases, meaning those which refer to the same concept in all context situations (called *semantic definites*, e.g., “*the time*”, “*the weather*”), differ in their processing from those definite noun phrases requiring introduction in the preceding context (called *pragmatic definites*, e.g., “*the product*”, “*the clock*”). Their results show a greater N400 for pragmatic definites compared to semantic definites. She interprets this result to mean that to fulfill a pragmatic definite a speaker has to search for a specific referent in the given context. This search requirement means that higher cognitive demands are present in this situation compared to one in which a semantic definite can be processed by activating a permanent concept. The above-mentioned results suggest that one and the same presupposition trigger, in this case the definite determiner, might be differently processed depending on the semantic/pragmatic context in which it is embedded.

In sum, several studies (Domaneschi and Di Paola 2018; Haviland and Clark 1974) have revealed that processing an unfulfilled presupposition triggered by the definite determiner comes along with enhanced processing costs. This processing cost has been shown to start at the moment of, or shortly after, reading the critical NP containing a presupposition violation (Domaneschi and Di Paola 2018; Kirsten et al. 2014; Schneider et al. 2020). These enhanced processing costs occur regardless of whether the context provides no adequate referent for the definite determiner (Clifton 2013; Garnham et al. 1997) or whether the context is ambiguous, providing obstacles to a smooth reference process (see e.g., Altmann and Steedman 1988). Moreover, some studies have also addressed the question of potential processing differences between definite and indefinite determiners (Clifton 2013; Gernsbacher and Robertson 2002; Murphy 1984). Yet, the evidence remains inconclusive. On the one hand, some linguistic theories assume that the indefinite and definite determiners elicit different assumptions, and several ERP studies provide evidence for differences in the processing of the two types of determiners (e.g., Schumacher 2009). On the other hand, experimental studies have shown similar unfulfillment effects for the two determiners (Clifton 2013; Kirsten et al. 2014). Hence, although the above-mentioned studies have provided valuable insights concerning presupposition processing at a cognitive level, the contribution of different cognitive processes on processing presupposition violations and their impact on processing different determiner types is far from clear.

The aim of the present study was to shed further light on the cognitive processing of presupposition triggers and, specifically, on the processing of the definite determiner and the indefinite determiner. To this end, we conducted four experiments using the word-by-word self-paced reading paradigm (see Tiemann et al. 2011). This paradigm can reveal insights into the overall time course of presupposition processing by measuring the reading time for every sentence element following the critical NP until the end of the sentence. Furthermore, by using different context situations for the same trigger, we aimed to get more insight into the question of whether the time point of presupposition processing is adaptive within a given context or if, in contrast, its variation depends solely on trigger type. In order to investigate these questions, we defined a critical word (wcrit) at which the fulfillment or non-fulfillment of the triggered presupposition becomes recognizable for the reader and placed it at different points in time within sentences. We expect that the reading time prolongations due to higher cognitive processing demands in the case of a violation of the presupposition should become evident while reading the critical word. Moreover, we forced participants to process the sentences at a semantic level (cf. Clifton 2013) and collected acceptability judgments. These judgments allowed to estimate how strongly participants experienced the presupposition violations and to get an impression of whether they might have accommodated the meaning of a sentence. The combination of recording acceptance judgments and reading times allows us to monitor potential changes in sentence interpretation and acceptability, which might be due to enhanced processing demands during sentence reading or which might follow when sentence reading had been finished.

In Experiment 1, we investigated the processing time courses of the definite and indefinite determiners by constructing fulfilled and unfulfilled discourse situations for each trigger type. Specifically, the unfulfilled discourse situation for the definite determiner violated the existence assumption, because we explicitly negated the existence of an object in the discourse context which is presupposed by the definite determiner. The unfulfilled discourse situation for the indefinite determiner violated the novelty assumption because we used the indefinite even though one of the indefinite entities had already been introduced in the context. In Experiment 2, we investigated the violation of the uniqueness assumption of the definite determiner by introducing in the context more than one potential referent for the definite. We studied the violation of the anti-uniqueness assumption of the indefinite determiner in this experiment by introducing only one entity in the context. In Experiment 3 and 4, we focused on the processing of the definite determiner in different contextual situations and investigated when during sentence reading participants processed the presupposition triggered by the definite determiner. In Experiment 3, we added non-restrictive versus restrictive subordinate sentences to the sentence containing the definite determiner to investigate whether participants used the restrictive sentence to establish an initially violated uniqueness of the referent. In Experiment 4, we introduced a group of discourse entities as well as a single discourse entity in the context sentence. The referential assignment triggered by the definite determiner in the test sentence could take place during the reading of the test sentence’s relative clause. We were interested in the question whether the possibility to resolve reference processes at a later time point during sentence reading would shift presupposition processing.

## Experiment 1

In the present experiment, we examined the existence presupposition associated with the definite determiner and the novelty assumption associated with the indefinite determiner. In order to investigate the time-course of presupposition processing, we presented contexts that explicitly included the existence of the critical noun (1) or explicitly expressed the non-existence of the critical noun (2). In a crossed design (see Altmann and Steedman 1988), context sentences were followed by test sentences using a definite (3b) or an indefinite (3a) NP. In case of the definite determiner, a retrieval of an antecedent from memory is not successful in the negated situation. In case of the indefinite determiner, the novelty assumption is violated if a single/novel entity had been introduced in the discourse. These two situations should cause processing difficulties resulting in prolonged reading times.

## Method

### Participants

A sample of 60 native speakers of German participated in this experiment. This study and the following ones were compliant with the ethics code of the World Medical Association (Declaration of Helsinki). Before the experiment, participants were informed about the duration of the experiment and they were told that they could interrupt the experimental session or finish the experiment whenever they feel uncomfortably. All of them gave written informed consent to participate in this experiment. We excluded seven of them because of technical problems. The remaining sample of 53 participants (45 women; mean age = 22.6; age range = 19–41) were students from the University of Tübingen or from the general working population in Tübingen. All participants had normal or corrected-to-normal vision. They were paid or received course credit for participation.

### Material

For the experiment, we created 48 sets of context-test sentences. Every set consisted of two different context sentences and two different test sentences (i.e., 192 context-test sentence pairs). One test sentence of a set included the definite determiner *der/die/das* (‘the’), see (3a), to trigger the existence presupposition of an NP. The need to search for a referent for the existence presupposition was enhanced by using a verbal phrase, which indicated a change of an existing discourse entity, such as *wash*, *paint*, *enlarge*, *glue*, or *configure*. The second type of test sentences contained the indefinite determiner *ein/eine* (‘a’) see (3b), and established a new discourse entity by using verbal phrases such as *build*, *buy*, *order* or *plant*. We joined the two types of test sentences with context sentences that established the existence presupposition either as true (1) as the NP was presented with the quantifier *ein/eine* (‘a’) or as unfulfilled (2) as the existence of the NP was negated by *kein/keine* (‘has no’). Each test sentence was combined with each context sentence. This combination resulted in different fulfillment conditions for the two types of test sentences: Test sentence 3a matched with context sentence 1, and it mismatched with context sentence 2. The reverse was true for test sentence 3b, containing the indefinite determiner: the negated context (2) provided the fulfilled condition as it fulfills the novelty assumption of the indefinite determiner, but violates it when existence of the discourse entity has already been established (1). A test sentences always consisted of nine words, the determiner occurred at the third, and the noun as a repetition of the noun of the context sentence at the fourth position and the critical verb (wcrit) at the fifth position. The following sentences constitute an example of an experimental set.

**Table:** 

(1)	Simon hat ein Vogelhaus, das Vögeln als Futterstelle im Winter dienen kann.
	*Simon has a birdhouse that birds as feeding/place in/the winter serve can.*
	‘Simon has a birdhouse that can serve as a feeding place for birds in winter.’
(2)	Simon hat kein Vogelhaus, das Vögeln als Futterstelle im Winter dienen kann.
	*Simon has no birdhouse that birds as feeding/place in/the winter serve can.*
	‘Simon does not have a birdhouse that can serve as a feeding place for birds in winter.’
(3a)	Während Simon das Vogelhaus streicht, schmiedet er einen Plan.
	*While Simon the birdhouse paints, makes he a plan.*
	‘While Simon paints the birdhouse, he makes a plan.’
(3b)	Während Simon ein Vogelhaus baut, schmiedet er einen Plan.
	*While Simon a birdhouse builds, makes he a plan.*
	‘While Simon builds a birdhouse, he makes a plan.’

To avoid response strategies and to mask the purpose of the experiment, 48 filler sentence sets (i.e., 192 filler trials) were designed in a similar way to the test material except that the filler sentences were semantically well-formed and contained neither an unfulfilled presupposition of the definite determiner nor an inappropriate use of the indefinite determiner. Definite and indefinite NP occurred equally often in the filler sentences, and the sentence structure of the filler sentences was analogous to the test sentences (e.g., *Simon has no workday and a little time to read. While Simon is reading an animal magazine, he's making up a story*). The filler sentences were randomly intermixed in the experimental material. To avoid memory strategies, immediate repetitions of sentences of the same set were minimized by arranging the stimulus material in four presentation blocks. Specifically, each possible test sentence pair of one experimental sentence set appeared in one block. The order of these blocks was balanced over participants according to a Latin square. Each participant worked on all sentence sets organized in the four blocks.^1^ Eight practice sentence pairs similar to the experimental sentences were constructed to familiarize participants with the procedure. Moreover, for each of the four protagonists of the discourse sentences an introducing global context was given at the beginning of the experiment. For example, the information participants read concerning one of the protagonists, Simon, was *“Simon has just built a house and still wants to finish a few things. He does a lot by himself and has to call a specialist for only a few things. He likes to meet friends for a beer or to invite them over to his place. He also likes to go to the theater. Simon is currently single. He works a lot and has a good job that he likes*.” The global context information was presented in the form of a text describing the four protagonists in sections. The text was presented on screen for all four protagonists before participants started to read the context-test-sentence pairs. Participants were told that this text introduces the protagonists and serves to get to know them and their habits. There was no mention of whether or how the global context relates to the later sentences. The same protagonists occurred in the test sentences and in the filler sentences. A different global context was designed for the practice trials and the protagonists mentioned in the practice trials.

To gain insight into the subjective experience of the different conditions, participants were asked to judge the content-related fit between test sentence and context sentence. This acceptability judgment included the categories: 1 = very bad, 2 = rather bad, 3 = rather good, 4 = very good. To encourage participants to process the sentences attentively at a semantic level, we included 384 different yes/no comprehension questions (e.g., “*Did Simon build a bird house?*”). These were presented following each test sentence trial as well as each filler sentence trial. The comprehension questions referred to different parts of the test sentences as well as to different parts of the context sentences and required a “yes”-response in half of the cases and a “no”-response in the other half. The subject of the question varied because we did not want to draw participants` attention to a specific part of the context sentence or the test sentence. Since the questions were sometimes directly relevant for the presupposition content, we hoped they would force participants to be sensitive to it (cf. Swets et al. 2008). Responses were collected via an external keyboard consisting of six separate keys.

### Procedure and design

Participants sat comfortably in a sound-attenuated room. The stimulus material was presented on a computer screen in white on a blue background. First, participants read a global context that introduced the relevant discourse entities in the practice material. After they finished the practice trials, the global context for the experimental discourse was presented, followed by the experimental trials. Each trial began with the presentation of a warning signal for 600 ms followed by a context sentence, which was shown as a complete sentence in the middle of the computer screen. After participants had read the context sentence, they requested the first word of the test sentence by a button press. The test sentence was thus presented word-by-word serially in the center of the screen in a self-paced manner—participants requested each subsequent word by a button press. The last element of a sentence was a period which was presented alone after the last word of the sentence. After the test sentence, a rating prompt appeared on the screen, and participants rated the acceptability of the test sentence within the given context. At the end of each trial, a yes/no-question was presented. A new trial started as soon as the participants pressed the response button to the comprehension question. Each session lasted about two hours and included eight break times. The independent variables were the factors Fulfillment (fulfilled vs. unfulfilled) and Determiner (definite vs. indefinite). As dependent variables, we measured the acceptability judgment scaled from one to four. In addition, we collected reading times as an online measure of presupposition processing.

### Data analysis

Statistical analyses were conducted in SAS (Version 9.4, SAS Institute Inc.). To reduce the number of statistical tests, we restricted the reading time analyses to the word before the trigger (*t*−1) as a baseline measure, the trigger (*t*) itself, the first word after the trigger (*t* + 1), the critical word (wcrit), the words following the critical word (w6, w7, w8), and the final word (fw) of the sentences. Following a truncation method (Ulrich and Miller 1994), reading times shorter than 100 ms and longer than 1500 ms were considered outliers.^2^ Trials containing outliers were discarded from the analysis. Reading time for each word was then analyzed via separate linear mixed-effects models (LMEMs) using restricted maximum likelihood (REML) fitting. Since we hypothesized a priori that the presupposition effect might differ between the two determiners, we included both main effects of Determiner (definite, indefinite) and Fulfillment (fulfilled, unfulfilled) and their interaction as fixed effects in our models. To determine an appropriate random effects structure that provided an adequate balance between Type I error rate and power, we followed the recommendation by Barr et al. (2013; see also Matuschek et al. 2017). Accordingly, we fitted LMEMs containing the maximal random effects structure in our design (i.e., crossed random intercepts for subjects and context as well as crossed random slopes for the effect of presupposition and definiteness with respect to subjects and context). Since these maximal models mainly fulfilled the convergence criterion, we did not reduce the random effects structure as suggested by certain authors, such as Jaeger (2009). For evaluation of the significance of each fixed effect in our final models, we report Type III *F*-tests with Satterthwaite-approximated degrees of freedom (see Luke 2017). This approach in combination with REML fitting has been shown to be superior to more conventional approaches (e.g., likelihood ratio tests or the *t*-as-*z*-approach) in terms of the Type I error rate (Luke 2017; see also Singmann and Kellen 2019). For each fixed effect, we report *F*-values (denoted as *F*_Sat_ to indicate that degrees of freedom are Satterthwaite-approximated), the corresponding degrees of freedom for the nominator and denominator, as well as the corresponding *p*-values.

## Results

Trials with RT outliers (5.37% of all trials) were discarded from the analysis. On average, participants answered 75.7% (range: 63.6–89.1%) of the comprehension questions correctly. 

### Acceptability judgments

**Fig. 1:**
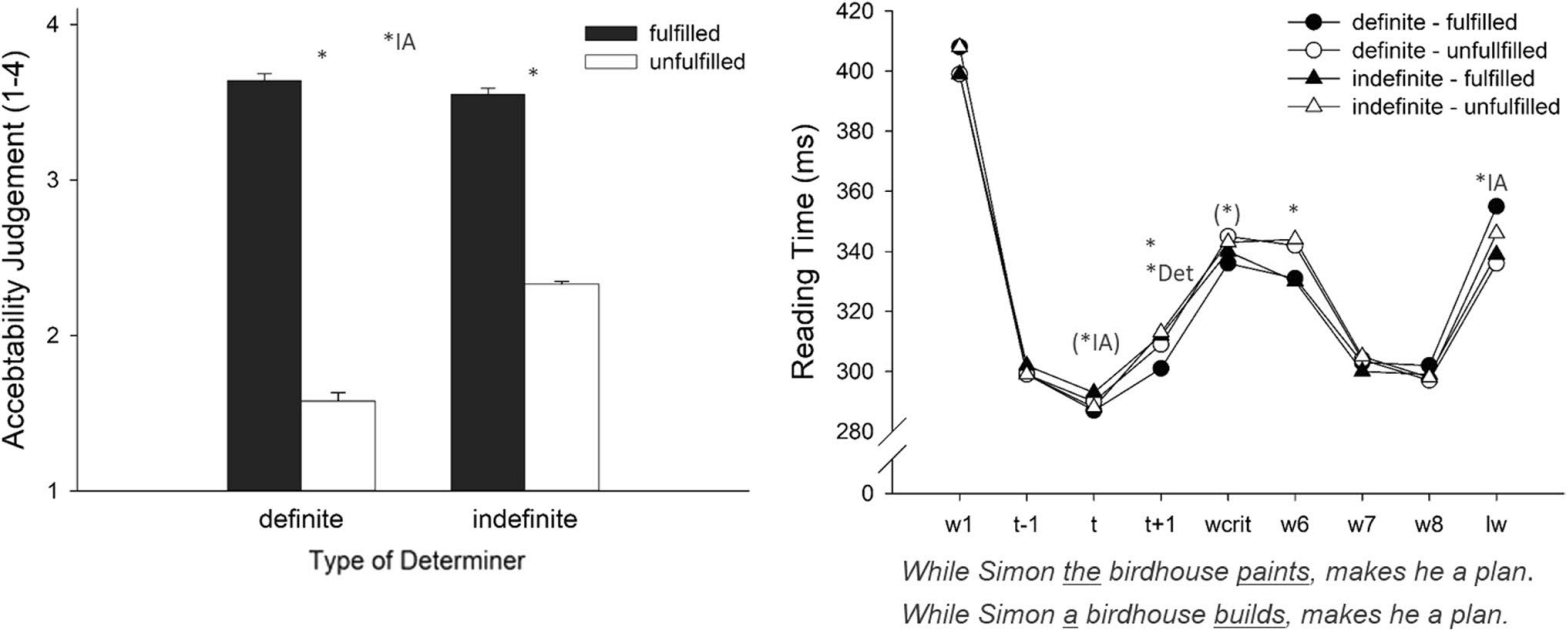
Acceptability judgments and reading times for sentences containing the definite and the indefinite determiner depending on fulfillment condition. Left panel: Mean acceptability values for target sentences. Right panel: Reading times (ms) for the words of interest (w = word, t = trigger, wcrit = critical word, lw = last word). The asterisks mark significant differences between conditions, and the asterisks in parentheses mark a tendency (* = Effect of Fulfillment, *Det = Effect of Determiner, *IA = interaction between Fulfillment and Determiner). The error bars shown for the acceptability judgments denote the standard error of the mean computed according to a method proposed by Cousineau (2005) for within-participants designs. We refrained from showing error bars for reading times for the sake of visual clarity

The acceptability judgments for the definite determiner and the indefinite determiner are presented in Fig. 1 (left panel). The LMEM analysis revealed that fulfilled sentences were judged to be more acceptable (*M* = 3.60) than unfulfilled sentences (*M* = 1.96), *F*_Sat_(1, 58) = 424.89, *p* < 0.001. There was a main effect of Determiner (*M*_indefinite_ = 2.94 vs. *M*_definite_ = 2.61), *F*_Sat_ (1, 141) = 79.90, *p* < 0.001, but this effect was qualified by a strong interaction between the two factors, *F*_Sat_ (1, 141) = 133.52, *p* < 0.001, reflecting the result that the unfulfillment effect was larger for sentences containing the definite determiner than for those containing the indefinite determiner.

### Reading times

Reading times for each word are shown in Fig. 1 (right panel). Overall, word reading times within the sentence show three peaks: they were high at the beginning of the sentences, increased again at the time point at which the trigger was presented, and increased a third time at the end of the sentence. For the word before the trigger (*t*−1), the LMEM analysis revealed no main effects of Determiner, Fulfillment, or an interaction of the two factors, all *F*_Sat_ < 0.38,* p*s > 0.54, indicating no processing differences before the critical presupposition trigger occurred in the sentence. For the trigger (t) itself, there was also no main effect, all *F*_Sat_ < 1.16,* p*s > 0.28, and a weak tendency toward an interaction between Fulfillment and Determiner, *F*_Sat_(1, 187) = 2.89, *p* = 0.09. A main effect of Fulfillment (*M*_fulfilled_ = 307 ms vs. *M*_unfulfilled_ = 311 ms) showed up in the word following the trigger (*t* + 1), *F*_Sat_(1, 95.1) = 4.00, *p* = 0.048, and there was a main effect of Determiner, *F*_Sat_(1, 41.6) = 6.76, *p* = 0.013, indicating longer reading times for sentences containing an indefinite determiner (*M* = 313 ms) compared to those containing a definite determiner (*M* = 305 ms). There was no interaction between the two factors, *F*_Sat_(1, 95.1) = 1.71, *p* = 0.19. For the critical word, there was a tendency toward an effect of Fulfillment (*M*_fulfilled_ = 338 ms vs. *M*_unfulfilled_ = 344 ms), *F*_Sat_(1, 46.9) = 4.00, *p* = 0.051, but no effect of Determiner or interaction, *F*_Sat_ < 1.20, *p* > 0.27. For the third word following the trigger (w6), there was a main effect of Fulfillment (*M*_fulfilled_ = 331 ms vs. *M*_unfulfilled_ = 343 ms), *F*_Sat_(1, 94.1) = 17.18, *p* < 0.001, but no effect of Determiner and no interaction, *F*_Sat_ < 0.17, *p* > 0.68. For the seventh word (w7) and the eighth word (w8), there was no effect, all *F*_Sat_ < 1.45,* p*s > 0.23. For the last word (lw), there was no main effect of Fulfillment, *F*_Sat_(1, 51.3) = 2.25, *p* = 0.14, or of Determiner, *F*_Sat_(1, 38.9) = 0.27, *p* = 0.61, but a strong interaction between Fulfillment and Determiner, *F*_Sat_(1, 94.3) = 16.69, *p* < 0.001.

## Discussion

The main aim of the present experiment was to examine the time-course of cognitive processing the existence presupposition and the novelty presupposition associated with the definite and the indefinite determiner, respectively. As expected, subjects rated sentences whose presuppositions were violated lower in acceptability than sentences whose presuppositions were fulfilled. However, the interaction between experimental factors on acceptability judgments shows that the unfulfillment effect differed between the two determiners. As outlined above, this difference between the determiners was expected based on theoretical considerations. The relatively high acceptability judgments for the unfulfilled sentences with indefinite determiners suggests that a violation of the indefinite determiner’s presupposition can be repaired in certain cases but that this “repair” process was not possible (at least in most cases) for the unfulfilled presupposition of the definite determiner. More specifically, in case of the infelicitous use of the indefinite, participants might re-interpret the test sentence by assuming that an entity other than the one introduced in the context was meant. In this sense, Simon builds another birdhouse in addition to the one he already has. Even though the indefinite determiner might not be used in an ideal manner in the unfulfilled sentence, the described form of accommodation might lead to an acceptable meaning of some sentences. Such a “repair” process is not possible for the definite determiner in the negated context, which leaves no room for a re-interpretation and thus is judged to be rather unacceptable. Except for the last word in the sentence, reading times showed comparable unfulfillment effects for the two determiners starting at *T* + 1. Thus, the different fulfillment effects for the two determiners that are present in the acceptability judgments are not accompanied by essential differences in fulfillment effects during the reading of the test sentence. We come back to this result in the General Discussion. Taken together, the results of Experiment 1 revealed that the word-by-word self-paced reading paradigm is a useful tool to track the time-course of presupposition processing.

## Experiment 2

As discussed in the Introduction, the definite determiner includes not only the existence presupposition, but additionally presupposes the uniqueness of a referenced entity (Heim 1982; Krahmer 1998; Strawson 1950). The introduction of more than one entity would cause difficulties for the reference process, because it would not be clear which of the entities is meant. In Experiment 2, we investigated what happens when the uniqueness presupposition of the definite is not fulfilled by introducing more than one potential referent in the context. As in Experiment 1, we compared these unfulfillment effects with those resulting from violations of assumptions bound to the indefinite determiner—in this experimental context, the anti-uniqueness assumption (Alonso-Ovalle et al. 2009; Hawkins 1991; Heim 1991).

## Method

### Participants

A new sample of 50 native speakers of German (37 women; mean age = 22.14; age range = 18–41) participated in this experiment. Most of them were students from the University of Tübingen; a few were employed adults from the general population. They had normal or corrected-to-normal vision. They were paid or received course credit for participation. No participant was excluded.

### Material

Eighty sets of experimental sentences very similar to the experimental sentences used by Schumacher (2009, see also Burkhardt 2006; Kirsten et al. 2014) were constructed. Each set consisted of two types of context sentences and two types of test sentences, with all possible combinations of the four leading to a total of 320 context-test sentence pairs. An example of such a sentence set is given below. One test sentence of a set contained the definite determiner *der/die/das* (‘the’), see (6a), to trigger the uniqueness presupposition of a NP. The test sentences containing the definite determiner were joined with context sentences that established the uniqueness presupposition either as true (4) or as unfulfilled (5). Specifically, in the fulfilled condition, the NP was presented with the quantifier *ein/eine* (‘a’) and in the unfulfilled condition, the NP in the context sentences was presented with quantifiers like *einige* (‘some’), *verschiedene* (‘several’), *viele* (‘many’) etc. For the test sentences that contained the indefinite determiner, the alternative context sentences constituted the fulfilled condition. Specifically, for test sentences containing *ein/eine* (‘a’), see (6b), context sentence (5) met the indefinite determiner`s assumption of many potential referents and thus this context-test sentence pair was termed fulfilled condition. When test sentences containing the indefinite determiner (6b) were combined with context sentences introducing only a single referent (4), the anti-uniqueness assumption of the indefinite determiner was violated and thus this combination was termed the unfulfilled condition. To prevent monotony and analogous to the study of Schuhmacher (2009; see also Kirsten 2014), in half of the sets the critical noun of the test sentence was a repetition of a noun in the context sentence (e.g., polar bear–polar bear), while in the other half it was a synonym (e.g., party member–politician).

**Table:** 

(4)	Antje war gestern im Zoo in Düsseldorf und besuchte einen Eisbären im Bärengehege.
	*Antje was yesterday in/the Zoo in Düsseldorf and visited a polar/bear in/the bear/enclosure.*
	‘Antje was in the Düsseldorf Zoo yesterday and visited a polar bear in the bear enclosure.’
(5)	Antje war gestern im Zoo in Düsseldorf und besuchte einige Eisbären im Bärengehege.
	*Antje was yesterday in/the Zoo in Düsseldorf and visited several polar/bears in/the bear/enclosure.*
	‘Antje was in the Düsseldorf Zoo yesterday and visited several polar bears in the bear enclosure.’
(6a)	Antje beobachtete, dass der Eisbär sehr aggressiv war.
	*Antje noticed that the polar/bear very aggressive was.*
	‘Antje noticed that the polar bear was very aggressive.’
(6b)	Antje beobachtete, dass ein Eisbär sehr aggressiv war.
	*Antje noticed that a polar/bear very aggressive was.*
	‘Antje noticed that a polar bear was very aggressive.’

The determiner was always the fourth word in each test sentence and each test sentence consisted of eight words in total. We included forty filler sentence sets (i.e., 160 context-test sentence pairs), designed in a way similar to the test material with the exception that they were semantically well-formed and contained neither an unfulfilled presupposition of the definite determiner nor an inappropriate use of the indefinite determiner (e.g., *Antje was at an art auction in Mannheim last week and looked at many pictures. She was very fond of a landscape drawing and bought it*.) Definite and indefinite NPs occurred equally often in the filler sentences. The presentation mode of the sentences was analogous to Experiment 1. We included 480 yes/no comprehension questions. As in Experiment 1, a global context text presented before the experimental reading time part introduced six protagonists which were mentioned in the test sentences as well as in the filler sentences.

### Procedure and design

The procedure and the statistical analyses were configured analogously to Experiment 1. The experiment included the factor Fulfillment (fulfilled vs. unfulfilled) and Determiner (definite vs. indefinite).

## Results

Altogether, 3.44% of the trials were removed as outliers (reading times < 100 ms and > 1500 ms). On average, participants answered 91.5% (range: 80.3–97.2%) of the comprehension questions correctly.

### Acceptability judgments

**Fig. 2:**
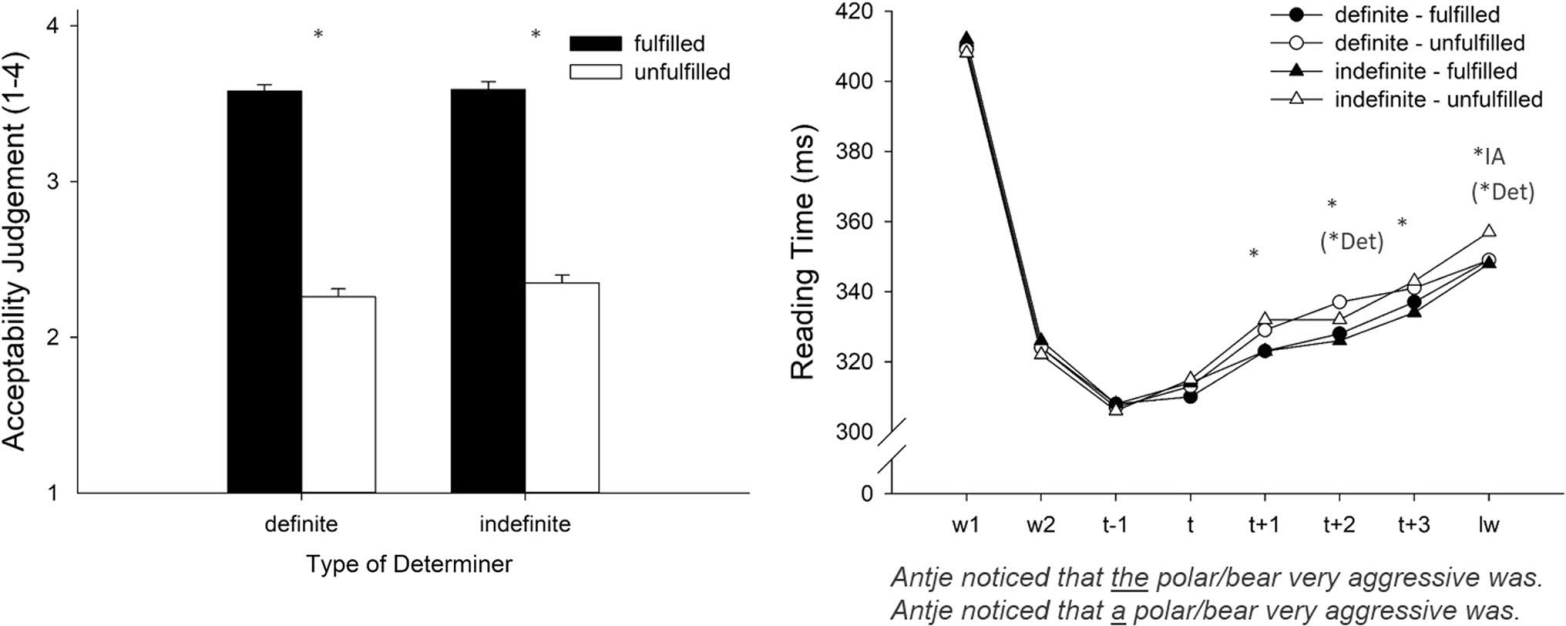
Acceptability judgments and reading times for sentences containing the definite and the indefinite determiner depending on fulfillment condition. Left panel: Mean acceptability values for target sentences. Right panel: Reading times (ms) for the words of interest (w = word, t = trigger, lw = last word). Note that in this Experiment, the trigger itself is the critical word. The asterisks mark significant differences, and the asterisks in parentheses mark a tendency (* = Effect of Fulfillment, *Det = Effect of Determiner, *IA = interaction between Fulfillment and Determiner)

The acceptability judgments for the definite determiner and the indefinite determiner are presented in Fig. 2 (left panel). Fulfilled sentences were judged to be more acceptable (*M* = 3.58) than unfulfilled sentences (*M* = 2.30), *F*_Sat_(1, 137) = 191.28, *p* < 0.001. There was no effect of Determiner, *F*_Sat_(1, 195) = 2.23, *p* = 0.14, nor was there an interaction between the two factors, *F*_Sat_(1, 195) = 1.76, *p* = 0.19.

### Reading times

Reading times for sentences containing the definite or the indefinite determiner and depending on Fulfillment condition are shown in Fig. 2 (right panel). For the word before the trigger (*t*−1), the analysis revealed no effect of either of the two factors, and there was no interaction between them, all *F*_Sat_ < 0.33, *p*s > 0.57. For the trigger (t) itself, there were again no effects, all *F*_Sat_ < 1.63, *p*s > 0.20. A main effect of Fulfillment (*M*_fulfilled_ = 323 ms vs. *M*_unfulfilled_ = 331 ms) showed up in the word following the trigger (*t* + 1), *F*_Sat_(1, 78.7) = 9.91, *p* = 0.002, but no other effects approached significance, all *F*_Sat_ < 0.7, *p*s > 0.44. There was also a main effect of Fulfillment (*M*_fulfilled_ = 327 ms vs. *M*_unfulfilled_ = 335 ms) for the second word following the trigger (*t* + 2), *F*_Sat_(1, 51.3) = 9.87, *p* = 0.004. In addition, there was a tendency toward a main effect of Determiner, *F*_Sat_(1, 158) = 3.08, *p* = 0.081. The interaction between the two factors was not significant, *F*_Sat_(1, 158) = 0.18, *p* = 0.67. The main effect of Fulfillment (*M*_fulfilled_ = 335 ms vs. *M*_unfulfilled_ = 342 ms) was still present at the third word following the trigger (t + 3), *F*_Sat_(1, 48.4) = 5.79, *p* = 0.020. No other effect reached significance level, all *F*_Sat_ < 1.19, all *p*s > 0.27. For the last word (lw), there was no main effect of Fulfillment, *F*_Sat_(1, 61.4) = 2.18, *p* = 0.15, and only a tendency toward an effect of Determiner, *F*_Sat_(1, 157) = 3.25, *p* = 0.07. There was an interaction between Fulfillment and Determiner, *F*_Sat_(1, 157) = 5.35, *p* = 0.022.

## Discussion

In this experiment, we investigated how violations of the uniqueness presupposition of the definite determiner and the anti-uniqueness of the indefinite determiner show up during the time-course of sentence reading. The acceptability ratings show that participants noticed the presupposition failure, and the unfulfillment effects were of similar strength for sentences containing the definite and the indefinite determiner. As in Experiment 1, the processing of the unfulfilled presuppositions was reflected in prolonged reading times for both determiners, starting shortly after reading the trigger (at *T* + 1). The time-course of this unfulfillment effect was very similar for sentences containing the two different triggers and it nearly lasted until sentence end. These late reading time effects at the end of sentences suggest that important processing steps take place at this time point. Since the sentence in this experiment was rather short, however, we cannot be sure whether these processes take place as a result of earlier processing steps and that this sequence just happens to end at the same time as the sentence, or whether these processes naturally happen at the end of the sentences. To investigate this question more specifically, we conducted two further experiments, in which we prolonged the test sentences.

## Experiment 3

In this experiment, we examined the time course of presupposition processing when the interpretation of the correctness of usage of the definite determiner was shifted away from the presentation of the presupposition trigger. Moreover, we prolonged the test sentences to capture hints of different cognitive processes potentially showing up in late reading time differences between fulfilled and unfulfilled conditions. We generated main sentences, which contained the definite NP, and these were followed either by a relative clause which further specified the NP and thus creates uniqueness (restrictive relative clause), or by a subordinate clause, which added some temporal information, and this information was not related to the NP. We assumed that these subordinate clauses could cause participants to revise the initially inappropriate use of the definite determiner in the context of several possible referents. Specifically, in a context setting introducing several possible referents for the NP of interest (8), the uniqueness presupposition was fulfilled by the restrictive relative clause (9a), but not by the temporal subordinate clause (9b). On the other hand, in a context in which only one discourse entity was introduced (7), the test sentence containing the restrictive relative clause was possible but maybe rather unusual, and the one containing the temporal subordinate clause was appropriate. We expected that the unfulfillment effect should be temporally shifted to the critical word, that is, the point at which participants could decide whether the subordinate clause was a restrictive or a non-restrictive one. This experiment was run concurrently with Experiment 4, that is, the same participants took part in the two experiments and the sentence material was intermixed.

## Method

### Participants

A new sample of 76 native speakers of German participated in this experiment. Due to technical problems, six participants had to be excluded. The remaining sample included 58 women (mean age = 24.44; age range = 18–50). Most of the participants were students from the University of Tübingen, and the rest were employed and from the general population. They had normal or corrected-to-normal vision. They were paid or received course credit for participation.

### Material

In total, 40 sets of context sets and test sets were created for the experiment (i.e., 160 trials). As in the previous studies, each set consisted of two different context sentences and two different test sentences. In this experiment, we focused solely on the definite determiner. The test sentences were combined with context sentences either mentioning a single referent (singular context) verifying the uniqueness presupposition of the definite determiner, or with a context mentioning many potential referents (plural context), initially violating the uniqueness presupposition. As in the previous studies, we collected acceptability judgments and answers for comprehension questions. The critical NP of the context sentence was—as in Experiment 2—presented either with the quantifier *ein/eine* (‘a’) or with quantifiers like *einige* (‘some’), *verschiedene* (‘several’), *viele* (‘many’). An example of one sentence set is given below.

**Table:** 

(7)	Antje war gestern im Zoo in Düsseldorf und besuchte einen Eisbären im Bärengehege.
	*Antje was yesterday in/the Zoo in Düsseldorf and visited a polar/bear in/the bear/enclosure.*
	‘Antje was in the Düsseldorf Zoo yesterday and visited a polar bear in the bear enclosure.’
(8)	Antje war gestern im Zoo in Düsseldorf und besuchte einige Eisbären im Bärengehege.
	*Antje was yesterday in/the Zoo in Düsseldorf and visited several polar/bears in/the bear/enclosure.*
	‘Antje was in the Düsseldorf Zoo yesterday and visited several polar bears in the bear enclosure.’
(9a)	Antje beobachtete, dass der Eisbär laut brummte, welcher einen braunen Fleck hatte.
	*Antje noticed that the polar/bear loudly growled, which a brown spot had.*
	‘Antje noticed that the polar bear loudly growled, which had a brown spot.’
(9b)	Antje beobachtete, dass der Eisbär laut brummte, als ein Besucher heftig hustete.
	*Antje noticed that the polar/bear loudly growled, when a visitor heavily coughed. *
	‘Antje noticed, that the polar bear loudly growled, when a visitor heavily coughed.’

A test sentence consisted of twelve words, and the determiner occurred at the fourth word in the test sentence. The subordinate clause started with the critical word at the eighth position. At this time point at reading, participants could evaluate the appropriate use of the definite determiner concerning its uniqueness presupposition. As in the previous studies, we included 160 yes/no comprehension questions and participants read a global context at the beginning of the experiment which introduces the six protagonists which were mentioned in the test sentences.

### Procedure and design

The procedure and the statistical analyses were configured analogously to Experiment 1 and Experiment 2. Experiment 3 included the factor Context (singular vs. plural context) and Sentence type (which vs. when).

## Results

Altogether, 4.35% of the trials were removed as outliers (reading times < 100 ms and > 1500 ms). On average, participants answered 88.1% (range: 96.3–54.5%) of the comprehension questions correctly.

### Acceptability judgments

**Fig. 3:**
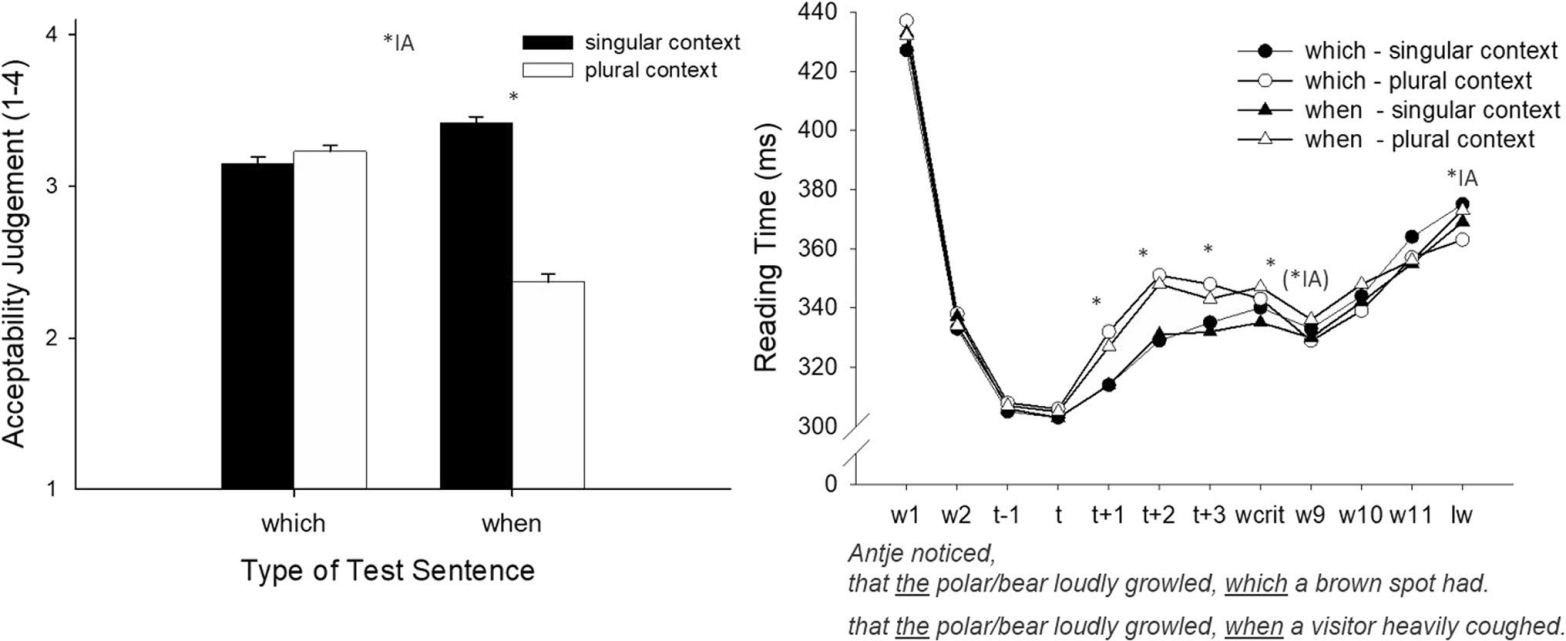
Acceptability judgments and reading times for the different sentence types (which-sentences and when-sentences) depending on context condition. Left panel: Mean acceptability values for target sentences. Right panel: Reading times (ms) for the words of interest (w = word, t = trigger, wcrit = critical word, lw = last word). The asterisks mark significant differences, and the asterisks in parentheses mark a tendency (* = Effect of Context, *Type = Effect of Sentence Type [which vs. when], *IA = interaction between Context and Sentence Type)

The acceptability judgments for the different sentence types are presented in Fig. 3 (left panel). Singular context sentences (*M* = 3.29) were judged to be more acceptable than plural context sentences (*M* = 2.80), *F*_Sat_(1, 105) = 65.44, *p* < 0.001. In addition, which-sentences (*M* = 3.19) were judged to be more acceptable than when-sentences (*M* = 2.90), *F*_Sat_(1, 169) = 22.86, *p* < 0.001. These main effects, however, were qualified by a strong interaction between the two factors, *F*_Sat_(1, 162) = 103.66, *p* < 0.001 indicating differences in the acceptability judgments for when-sentences and which-sentences depending on singular versus plural context.

### Reading times

Reading times for the which-sentences and the when-sentences are shown in Fig. 3 (right panel).

For the word before the trigger (*t*−1), the analysis revealed no main effects or interaction between the two factors, all *F*_Sat_ < 1.2, and all *p*s > 0.29. For the trigger (*t*) itself, there were no effects, all *F*_Sat_ < 1.87, all *p*s > 0.17. Context was significant for the word following the trigger (*t* + 1), *F*_Sat_(1, 67) = 4.23, *p* = 0.044, and revealed longer reading times for plural context sentences (*M*_plural context_ = 329 ms) than for singular context sentences (*M*_singular context_ = 314 ms). No other effect approached significance, all *F*_Sat_ < 1.3 and *p*s > 0.25. The context effect was still present for the second word following the trigger (*t* + 2), *F*_Sat_(1, 67.6) = 12.68, *p* < 0.001 (*M*_plural context_ = 349 ms vs. *M*_singular context_ = 330 ms), and there were no further effects, all *F*_Sat_ < 1.4, all *p*s > 0.23. The effect of Context continued to the third word following the trigger (*t* + 3), *F*_Sat_(1, 63.1) = 6.73, *p* = 0.012 (*M*_plural context_ = 346 ms vs. *M*_singular context_ = 333 ms). Again, there was no main effect of Sentence Type or an interaction between factors, all *F*_Sat_ < 2.57, all *p*s > 0.11. At the critical word (wcrit), reading times again showed an effect of Context, *F*_Sat_(1, 95.6) = 5.86, *p* = 0.017 (*M*_plural context_ = 344 ms vs. *M*_singular context_ = 338 ms), but no further effects, all *F*_Sat_ < 2.0, all *p*s > 0.17. For the ninth word (w9), there was a tendency toward an interaction *F*_Sat_(1, 94.5) = 3.14, *p* = 0.080, but no further main effects, all *F*s < 0.34, all *p*s > 0.56. For the tenth word (w10), and the eleventh word (w11) there was neither a main effect nor an interaction, all *F*s < 2.75, all *p*s > 0.10. For the last word (lw), there was an interaction, *F*_Sat_(1, 97.9) = 5.02, *p* = 0.027, but no main effects, all *F*s < 1.05, all *p*s > 0.30.

## Discussion

The first interesting result of Experiment 3 concerns the acceptability judgments. These show that the rating patterns differ fundamentally between the test sentences: the which-sentences were judged to be equally acceptable irrespective of context, whereas the when-sentences in the plural context were clearly judged to be less acceptable than the when-sentences in the singular context. The similar acceptability judgments for the which-sentences suggest that participants were able to make sense of the sentences independently of the uniqueness manipulation. The interpretation of the meaning, however, might differ depending on the type of context. Specifically, when the context introduced many discourse entities, the which-sentences were most probably interpreted as a clarification and restriction as to which one out of the mentioned discourse entities was meant, and by this, uniqueness was achieved. In the singular context condition, the which-sentences were most probably interpreted as an appositive relative clause that added further individual information to the single mentioned discourse entity. This interpretation explains why—after interpreting the relative clause—participants no longer experienced the initial mismatch between the definite determiner and the number of referents in the context sentence as infelicitous. In contrast to the which-sentences, participants experienced the when-sentences in the plural context as less acceptable than in the singular context. We expected this result since the when-sentences did not allow any restriction as to which out of the many discourse entities mentioned in the context was meant in the test sentence, the consequence being that the sentence contained a violation of the uniqueness presupposition of the definite determiner up to its end.

Even though the acceptability ratings essentially differ between which- and when-sentences, reading times revealed similarities for the two sentence types. First, reading times started to diverge shortly following the presentation of the trigger and prolonged reading times resulted in the plural context for which- as well as for when-sentences. This effect mirrors the mismatch between several discourse entities mentioned in the context and the definite determiner (i.e., “*the* polar bear”) used in the test sentence for both sentence types. We interpret this effect as a sign that participants might have a “default mode” for processing the definite determiner and that this mode requires an existing and unique referent. When the definite determiner was paired with a context introducing more than one referent for it, participants seemed to notice the initially inappropriate use of the definite determiner and invested cognitive resources in anticipation of a solution for the presupposition at a later time point at sentence reading. Second, the initial “mismatching effect” disappeared for both sentence types shortly after the subordinate sentence started with the presentation of the critical word. No further meaningful differences between context conditions and sentence types occurred while participants read the subordinate clause. We come back to the differing results for reading times and acceptability judgments in the General Discussion.

## Experiment 4

In Experiment 4, the context sentence contained a group of discourse entities as well as a single discourse entity. The reference to one of these possible discourse entity types was done by assigning these discourse entities either an agent or a patient role and referring to one of these reference possibilities by using the agent or the patient role in the test sentences. For example, in both the context sentence (10), and in the test sentence (12a) the single discourse entity was assigned an agent role (and the group of discourse entities a patient one) and thus, the test sentence was used in an appropriate way. The same was true in the sentence pairing which consistently assigned the single discourse entity a patient role (and an agent one to the group of discourse entities) in both sentences as in (11) and (12b). The other sentence-to-context-pairings contained violations of uniqueness as the single and plural agent and patient roles did not fit together. We expected that participants would experience the unfulfillment at the critical time point when they were able to clarify the patient versus agent role of the discourse entities depending on the voice (passive versus active) of the test sentence.

### Method

#### Participants

The same sample of 76 native speakers of German as in Experiment 3 participated in this experiment as the two experiments were run within the same experimental session.

### Material

In total, 40 sets of context sets and test sets were created for the experiment (i.e., 160 trials). As in the previous studies, each set consisted of two different context sentences and two different test sentences. As in Experiment 3, we focused solely on the definite determiner. The test sentences in this version consisted of a main clause and a relative subordinate clause, which determined the agent or patient role of the single discourse entity mentioned in the context sentence by using passive or active voice. Fulfillment in this situation was defined by the correspondence of the roles of the single protagonist in the context sentence and in the test sentence: A match was considered a fulfilled condition and a mismatch was considered an unfulfilled condition. The test sentence was twelve words long and the definite NP was presented at position six. The verb phrase allowing participants to judge the fulfillment of the presupposition occurred at the tenth position. Again 160 yes/no comprehension questions were constructed to fit these sentences. No global context was generated for this experiment because no recurring protagonists appeared.

**Table:** 

(10)	In der Mittagshitze der Prärie warnte ein Erdmännchen mehrere andere Erdmännchen vor einem Adler in der Luft und rannte anschließend schnell davon.
	*In the midday/heat of/the prairie warned a meerkat several other meerkats about an eagle in the air and ran then quickly away.*
	‘In the midday heat of the prairie, a meerkat warned several other meerkats about an eagle in the air and then quickly ran away.*’*
(11)	In der Mittagshitze der Prärie warnten mehrere Erdmännchen ein anderes Erdmännchen vor einem Adler in der Luft und rannten anschließend schnell davon.
	*In the midday/heat of/the prairie warned several meerkats another meerkat about an eagle in the air and ran then quickly away.*
	‘In the midday heat of the prairie, several meerkats warned another meerkat about an eagle in the air and then quickly ran away.*’*
(12a)	Im nächsten Frühling jedoch wurde das Erdmännchen, das gewarnt hatte, leider gefressen.
	*In/the next spring however was the meerkat that warned had unfortunately eaten.*
	‘Next spring, however, the meerkat that had warned unfortunately was eaten.’
(12b)	Im nächsten Frühling jedoch wurde das Erdmännchen, das gewarnt wurde, leider gefressen.
	*In/the next spring however was the meerkat that warned had/been unfortunately eaten.*
	‘Next spring, however, the meerkat that had been warned unfortunately was eaten.’

### Procedure and design

The procedure and the statistical analyses were configured analogously to Experiment 3 and included the factor Fulfillment (fulfilled vs. unfulfilled) and Sentence type (active vs. passive).

## Results

Altogether, 7.85% of the trials were removed as outliers (reading times < 100 ms and > 1500 ms). Participants answered 92.5% (range: 60.1–97.8%) of the comprehension questions correctly.

### Acceptability judgments

**Fig. 4:**
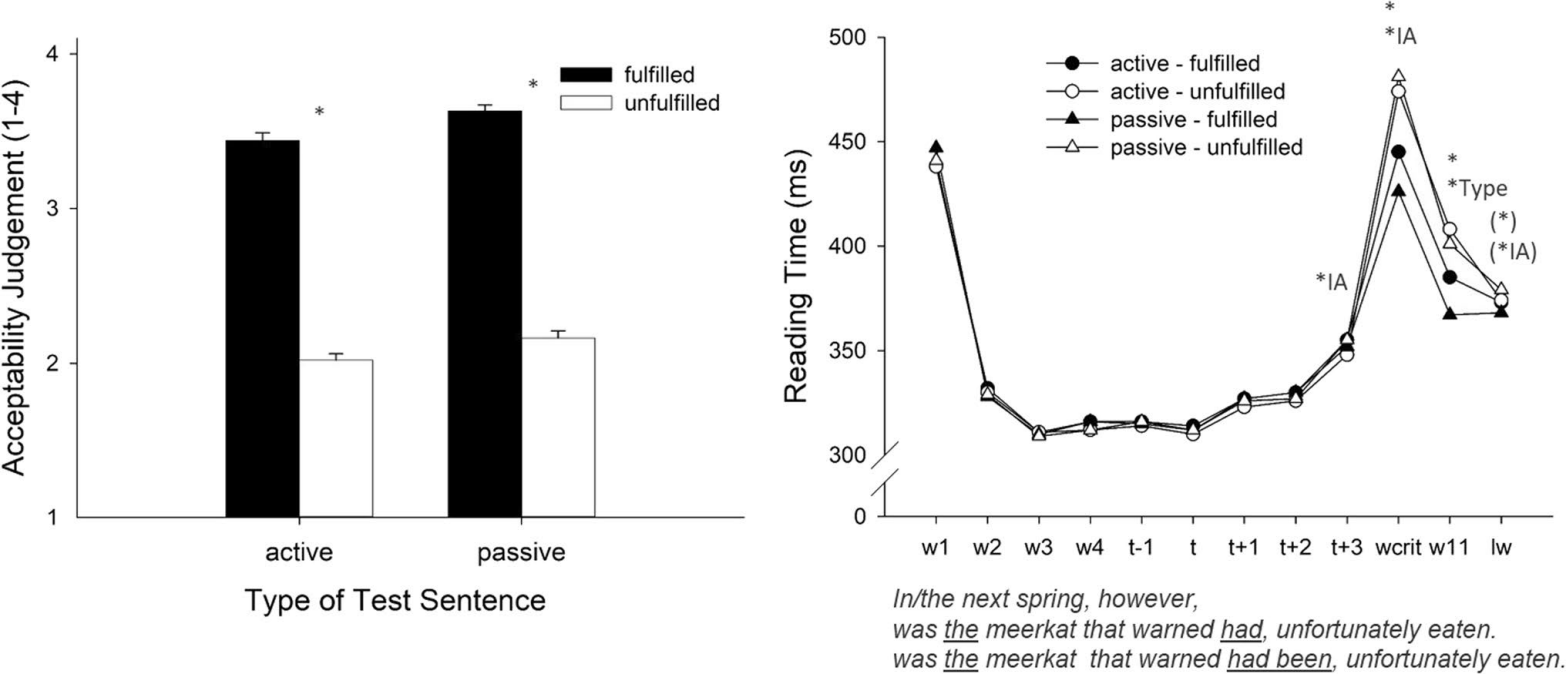
Acceptability judgments and reading times for the different sentence types (active and passive sentences) depending on fulfillment condition. Left panel: Mean acceptability values for target sentences. Right panel: Reading times (ms) for the words of interest (w = word, t = trigger, wcrit = critical word, lw = last word, remark: was/eaten corresponds to “gefressen,” i.e., a single word in German). The asterisks mark significant differences and the asterisks in parentheses mark tendencies toward effects (* = Effect of Fulfillment, *Type = Effect of Sentence Type [active vs. passive], *IA = interaction between Fulfillment and Sentence Type)

The acceptability judgments are presented in Fig. 4 (left panel). Fulfilled sentences (*M* = 3.53) were judged to be more acceptable than unfulfilled sentences (*M* = 2.09),* F*_Sat_(1, 142) = 365.91, *p* < 0.001. In addition, passive-sentences (*M* = 2.89) were judged to be more acceptable than active-sentences (*M* = 2.73),* F*_Sat_(1, 122) = 13.14, *p* < 0.001. There was no interaction between the two factors, *F*_Sat_(1, 33.2) = 1.38, *p* = 0.248.

### Reading times

Reading times are shown in Fig. 4 (right panel). Again, and in line with all other experiments, there was enhanced reading time for the first word in the sentence. Reading times increased slightly in the reading region following the presupposition trigger, but started to increase more strongly at the critical word. This impression is supported by the statistical analyses.

These revealed no effect for the word before the trigger (*t*−1), for the trigger (*t*) itself, the word following the trigger (*t* + 1), and for the second word following the trigger (*t* + 2), all *F*_Sat_ < 2.11, all *p*s > 0.15. An interaction between the two factors was present at the third word following the trigger (*t* + 3), *F*_Sat_(1, 77.1) = 4.64, *p* = 0.034, but there were no main effects of Fulfillment or Sentence type, all *F*_Sat_ < 0.36, all *p*s > 0.55. At the critical word (wcrit), there was a strong Fulfillment main effect (*M*_fulfilled_ = 436 ms vs. *M*_unfulfilled_ = 478 ms), *F*_Sat_(1, 69.9) = 16.69, *p* < 0.001. This main effect was accompanied by an interaction of Fulfillment and Sentence type, *F*_Sat_(1, 49.6) = 8.22, *p* = 0.006. The main effect of Sentence type was not significant, *F*_Sat_(1, 48.9) = 1.57, *p* = 0.217. The Fulfillment main effect was also present on word eleven (w11) (*M*_fulfilled_ = 376 ms vs. *M*_unfulfilled_ = 405 ms), *F*_Sat_(1, 55.7) = 49.89, *p* < 0.001, and there was an additional main effect of Sentence type, indicating longer reading times for the active-sentences (*M* = 396 ms) than for the passive-sentences (*M* = 384 ms), *F*_Sat_(1, 47.4) = 6.48, *p* = 0.014. The interaction between the two factors was not significant, *F*_Sat_(1, 46.6) = 1.97, *p* = 0.167. For the last word (lw), there was a tendency toward a main effect of Fulfillment, *F*_Sat_(1, 63.4) = 2.80, *p* = 0.099, and one toward an interaction, *F*_Sat_(1, 117) = 2.89, *p* = 0.092, but there was no a main effect of Sentence type, *F*_Sat_(1,117) = 0.05, *p* = 0.817.

## Discussion

The results of Experiment 4 are clear-cut. The acceptability ratings show a strong unfulfillment effect for the active sentences as well as for the passive sentences. Reading times showed that participants did not experience an initial presupposition violation in the unfulfilled situations when the definite determiner was presented. This result somehow differs from the results of Experiment 3, in which reading the definite determiner in the case of plural contexts immediately resulted in an increase in reading times due to an unclear reference situation. One explanation for the discrepancy between the experiments could be that, in Experiment 4, the context-sentences offer the possibility of a potentially suitable unique referent: they mentioned a single referent as well as a group of potential referents. Even though the reference problem in that situation cannot be solved at the time point when the trigger is presented and must be postponed to a later point in time, the presence of a potential unique referent in the context may inactivate the “default mode” described in the discussion of Experiment 3. Immediately at the critical word, the first signs of a presupposition violation occurred. This immediate and prolonged response in reading times shows that participants focused on this critical word to resolve the reference ambiguity.

## General discussion

The aim of the present study was to provide further insight into the cognitive processing of the definite determiner and the indefinite determiner concerning their role as presupposition triggers. To this end, we conducted four experiments in which we measured both acceptability judgments and word-by-word reading times to investigate the time-course of processing the definite and indefinite determiner. The acceptability judgments show that participants were aware of violated presuppositions, judging sentences containing an unfulfilled presupposition to be less acceptable than those containing a fulfilled presupposition. This result is in line with theories assuming that the inadequate use of presupposition triggers results in difficulties evaluating the meaning of a sentence (e.g., Heim and Kratzer 1998). There were, however, some (initially) unfulfilled sentences, which were judged relatively high in acceptability. This was the case for the sentences with the indefinite determiner in Experiment 1 and for the which-sentences in Experiment 3 (in which there was no difference in acceptability judgments between singular and plural context sentences). These high acceptability judgments for sentences containing an unfulfilled presupposition suggest that these sentences were re-interpreted and that their meaningfulness was “salvaged” in some cases by an accommodation process. What could such “repair” processes look like? For example, in case of the indefinite determiner triggering the novelty presupposition in Experiment 1, which was violated by the fact that the context sentences had already introduced the discourse entity, a re-interpretation process could be triggered by the belief that an *obligatory presupposition* extension such as “*another*” or “*further*” was left out in an unfulfilled indefinite sentence (see, e.g., Krifka 1999). Such kind of re-interpretation would result in the establishment of a new (and additional) discourse item for the indefinite. As mentioned in the discussion of Experiment 3, the interpretation of the which-sentence as clarification as to which one out of the mentioned discourse entities was meant or as an apposition of the referenced entity resulted in relative high acceptability ratings. Even the violated uniqueness presupposition of the definite determiner (Experiment 2 and Experiment 3 when sentences, plural context), did not show acceptability ratings that indicate a complete rejection. Thus, it is reasonable to assume that these violations might be accommodated at least to some extent. Such imperfect reference process could be the assumption that one out of the several entities that had been introduced was meant. In this sense, participants could repair the meaning of the sentence by re-interpreting the definite determiner as “*one of these*”. In sum, not only the different types of triggers (such as the type of determiner) have to be considered when thinking about cognitive processes underlying presupposition processing; the different presuppositions that are initiated by a specific trigger, like for example the existence presupposition and the uniqueness presupposition of the definite determiner must be considered as well. In general, the ratings afford some important clues to the nature of cognitive processes presumably involved in presupposition processing.

The reading time data also revealed several interesting aspects of presupposition processing. First, violated presuppositions incurred longer reading times. This result is consistent with those observed by several authors (Altmann and Steedman 1988; Clifton 2013; Domaneschi and Di Paola 2018; Garnham et al. 1997; Haviland and Clark 1974). We assume that this result mirrors an extended memory search and reference process and a subsequent evaluation phase in the unfulfilled case. These additional processes are not necessary if a trigger can be smoothly matched with a referent in the fulfilled condition. Second, in sentences in which the sentence structure did not facilitate a resolution of an initial presupposition violation (Experiments 1 and 2), longer reading times for unfulfilled sentences were present soon after the occurrence of the trigger. This result corresponds nicely to the study of Domaneschi and Di Paola (2018), who reported an unfulfillment effect on *T* + 1. It is also compatible to results obtained when the word “*also*” was used to trigger a presupposition (Schwarz 2007).

In contrast to these immediate effects, the unfulfillment effect in Experiment 4 (active and passive sentences) was shifted toward the end of the sentences. This overall pattern of results allows for further conclusions concerning the cognitive processes involved in presupposition processing. On the one hand, these results imply that a presupposition trigger does not automatically trigger an “unfulfillment reaction” if the sentence structure inherits a later verification possibility. For the specific presuppositions used in these sentences, this possibility came when the critical word defined whether the discourse entity of the test sentence had a unique referent in the context sentence. The reading time prolongation elicited by the determiner in Experiment 3, on the other hand, shows that a mismatch between the definite determiner and several referents in the context was immediately detected. We interpret this increase in reading times as hint that cognitive resources were recruited to attentively process the forthcoming sentence elements for presupposition solution. The results of the Experiments 3 and 4 together suggest that cognitive resources were distributed “on demand,” that is, depending on whether and when a solution is possible during sentence processing. This interpretation is compatible with Ferreira and colleagues’ idea of an explicit prediction of forthcoming comprehension processes or “preparedness” in language processing (Ferreira and Chantavarin 2018; see also Ferreira and Lower 2016). It is also well in line with other results and theoretical considerations in language processing according to which the *“…context influences the state of the language processing system before the bottom-up input is observed*.” (Kuperberg and Jaeger 2016, p. 2). Interpreted within this framework, participants in our study might have an idea of the sentence structure and expect an upcoming solution for the initially difficult sentence interpretation (caused by the mismatch of multiple referents for the definite determiner). To prepare for this, they may schedule cognitive resources. This preparedness may then facilitate interpretation when the critical sentence part (critical word) occurs, so that processing difficulties quickly disappear (Experiment 3). Furthermore, Experiment 4 suggests that this kind of preparedness for certain necessary processes allows readers to focus on critical sentence elements and ignore ambiguities that had occurred earlier, probably also leading to the facilitation of integration and understanding. In this sense, our results suggest that comprehension processes are not only retroactive and integrative, but that contextual expectations can lead to a bundling of cognitive resources. In sum, the observed time points of the unfulfillment effect in the four studies strengthen the observation that presupposition processing begins immediately when a trigger is encountered or as soon as a verification of the triggered presupposition is possible.

Regarding potential processing differences between the definite and indefinite determiner, the results of the present study are overall more compatible with the idea of more processing similarities than differences between the two determiners—at least during reading of the sentences as indexed by the word-by-word reading times in the present study. On the other hand, Experiment 1 clearly showed that the unfulfillment effect in the acceptability judgments differed between the definite and indefinite determiner: Despite no observable differences in reading times, unfulfilled presuppositions were rated to be more acceptable in case of the indefinite determiner than in case of the definite determiner. This particular result is noteworthy because it indicates that inferring the meaning of a sentence might not be a local, incremental process (i.e., during sentence reading) but can still be ongoing after information uptake (i.e., at the end of the sentence and/or afterward). Measuring acceptability judgments in addition to reading times may therefore be important to capture such post-sentence-reading processes.

Furthermore, from a more general perspective, the differences in the acceptability judgment imply that the two determiners have a different potential for accommodation processes and that this potential depends on the type of presupposition they carry and on the context in which they are embedded. Such a view may also help to understand why previous empirical studies revealed processing differences between determiners that went in different directions. Specifically, in some studies, it was reported that it takes longer to understand indefinite than definite references (Clifton 2013; Gernsbacher and Robertson 2002; Murphy 1984), which suggests that processing the indefinite determiner requires higher cognitive processing demands. The results of other studies, however, suggest the opposite, that the definite determiner presents higher processing demands (Anderson and Holcomb 2005; Schumacher 2009; Kirsten et al. 2014). The view that the specific type of presupposition a determiner carries has to be taken into account along with the type of determiner when examining the cognitive processes underlying presupposition processing is compatible with functional neuroimaging data showing differential results depending on determiner type and on ease of accommodation. For example, using functional magnetic resonance imaging (fMRI), Robertson et al. (2000) found that reading sentences with definite determiners was associated with stronger activation of right frontal areas than reading sentences with indefinite determiners. Sentences containing indefinite determiners were instead associated with enhanced neuronal activity in the *left* frontal hemisphere. The authors interpreted their results as evidence for a functional role of the right hemisphere in coherent discourse mapping, a process that is only required for definite determiner sentences. Moreover, in an fMRI study by Dietrich et al. (2019), it was shown that activations in some brain areas (i.e., supplementary motor cortex and basal ganglia) were modulated depending on the possibility of accommodating presupposition violations for the indefinite determiner. All these studies provide evidence that not only the trigger itself, but the specific embedding in different contexts as well as the sentence structures influence the cognitive processing demands of presuppositions.

Our findings support the assumptions described in the Introduction and expand knowledge upon cognitive processing of presuppositions by revealing some boundary conditions as well as necessary additions. On the basis of theoretical considerations, the reviewed experimental findings, and the present findings of this study, we propose the following working architecture of presupposition processing. First of all, usually the presupposition trigger serves as the starting point within a sentence where a reader initiates a reference to the established discourse context in order to check whether a presupposition is satisfied. We called this first step the *reference process and memory process* to account for the idea that, even in situations in which no referent was introduced in the context and a reference process is not successful, a memory search process is started which looks for a probable referent in memory. This process requires cognitive resources and thus prolongs reading times for triggers compared to most other sentence words (see e.g., Tiemann et al. 2011). Subsequently, an *evaluation process* might check whether the presupposition is satisfied in the context (e.g., Kirsten et al. 2014) or might be satisfied during later sentence reading. Importantly, the outcome and duration of this evaluation process might differ depending on the fulfillment condition: It is successful and can be rapidly finished in the fulfilled condition, as soon as the content of the presupposition can be verified. In contrast, the evaluation process needs to continue in the unfulfilled condition until the decision is reached that either the content of the presupposition cannot be guaranteed or, alternatively, that a re-interpretation of the discourse is necessary (Altmann and Steedmann 1988; Gundel et al. 1993). The results of the present study suggest that these processes take place during sentence reading shortly following the time point when participants read the trigger and/or when they can verify whether a presupposition is fulfilled or not at the critical word. The evaluation process might be followed by an *accommodation process* (see e.g., Beaver and Zeevat 2007; Stalnaker 1973), which, under some circumstances, inserts missing information into the discourse context to save a sentence from being meaningless (e.g., Lewis 1979; Kadmon 2001). As part of the accommodation process, we assume that there is an integration phase, which updates the context by adding new content or specifying existing content. We have characterized this phase as for example the insertion of a new entity into the context (Experiment 2), or as an update of focused information to the context (Experiment 3). We can only hypothesize that this phase requires extra cognitive resources following sentence reading and occurs most probably at the end of the cognitive processing chain that characterizes presupposition processing.

This characterization of the integration phase corresponds to Kintsch's *construction-integration model* (1988; for a formalized idea of a discourse representation and integration of presupposition knowledge in a discourse context, see Venhuizen et al. 2018). Specifically, Kintsch explicitly assumes the generation of different alternatives, which are either chosen and integrated or inhibited and rejected during the integration process. While, on the one hand, our results show that some of these processes start at trigger presentation, on the other hand, they also show that knowledge of the sentence structure and the expectation of certain information facilitates a focused processing of informative sentence components (Experiments 3 and 4). These findings emphasize the influence of expectations on language processing (Ferreira and Chantavarin 2018; Ferreira and Lower 2016; Kuperberg and Jaeger 2016) and nicely correspond to the results of Domaneschi et al. (2018; see also Domaneschi et al. 2014), who proposed that cognitive processes differ depending on the requirements for different presuppositions.

Although the proposed processing architecture integrates empirical results and theoretical considerations concerning presupposition processing, it obviously does not capture all linguistic theories and assumptions. For example, the definite determiner might sometimes be a part of an assertion rather than a presupposition trigger (e.g., Russell 1905). Moreover, the definite determiner may serve purposes other than indicating existence and uniqueness and through those purposes might influence cognitive processing in different ways. Specifically, the definite determiner has been considered to trigger familiarity (Heim 1982), salience (Lewis 1979), or anaphoricity (van der Sandt 1992) (see Krahmer 1998 for an overview). It would be interesting to investigate whether the processing of presupposed information held in short-term memory differs from information that is retrieved from long-term memory. It might also be interesting to investigate under which conditions people show relatively shallow processing (e.g., Clifton 2013) to reach a “*good-enough representation*” (Sanford and Graesser 2006) and when they instead attempt to find an unambiguous interpretation of the discourse content. It is likely that these different modes of processing, for example, the requirement to make an acceptability judgment, the specific contextual situation, or the sentence structure, induce differences in accommodation and integration behavior. It would be interesting to investigate these potential integration processes for which our acceptability ratings provide strong hints. Taken together, several open questions remain, which will have to be investigated to evaluate the general assumptions of presupposition processing.

In summary, the present study shows that reading times increase when a presupposition is not given in a context compared to a situation in which the context fulfills the presupposition. The time point of occurrence of reading time difference between a fulfilled and an unfulfilled condition was not fixed on the noun immediately following the definite or the indefinite determiner, but could also be shifted toward more distant words if they provided relevant information about whether a presupposition was fulfilled or not. Theoretically most important, the pattern of the fulfillment effects as indicated in acceptability judgments differed depending on which presupposition was violated by the different triggers. We assume that some cognitive processes associated with presupposition processing (reference processes and memory processes) occur during reading of a sentence, but that potential re-interpretation and integration processes might take place at a later time point following sentence reading.

## Data Availability

The data and the evaluation scripts will be provided on request.

## Appendix

See Tables 1, 2, 3, 4.

**Table 1:** Overview over the results of Experiment 1

Means (SEM)	Acceptability	W1	*T*−1	*T*	*T* + 1	Wcrit	W6	W7	W8	LW
*First block*										
Definite—fulfilled	3.54 (0.04)	446 (5.59)	345 (3.29)	333 (3.35)	328 (4.87)	419 (5.81)	414 (6.33)	365 (4.02)	369 (4.30)	466 (7.55)
Definite—unfulfilled	1.57 (0.05)	436 (6.13)	349 (3.67)	337 (3.09)	340 (4.55)	422 (5.88)	435 (7.42)	361 (3.87)	350 (3.54)	428 (6.23)
Indefinite—fulfilled	3.47 (0.04)	431 (5.95)	354 (3.53)	341 (3.56)	335 (3.32)	421 (7.07)	397 (6.00)	360 (3.96)	359 (3.72)	438 (6.96)
Indefinite—unfulfilled	2.36 (0.04)	434 (4.72)	351 (3.46)	333 (3.29)	343 (4.16)	434 (5.45)	438 (8.04)	372 (3.97)	361 (4.47)	452 (8.56)

Factor	*F*	*p*	*F*	*p*	*F*	*p*	*F*	*p*	*F*	*p*	*F*	*p*	*F*	*p*	*F*	*p*	*F*	*p*	*F*	*p*
Determiner	**61.75**	** < *****.001***	1.80	*.187*	1.20	*.273*	0.33	*.566*	1.14	*.291*	0.83	*.367*	0.31	*.579*	0.26	*.613*	0.01	*.933*	0.02	*.888*
Fulfillment	**400.74**	** < *****.001***	0.16	*.691*	0.24	*.621*	0.07	*.795*	3.52	*.071*	1.50	*.224*	**21.33**	** < *****.001***	0.69	*.410*	3.03	*.089*	3.44	*.072*
Det x Ful	**94.21**	** < *****.001***	0.77	*.381*	0.90	*.343*	1.52	*.218*	0.04	*.835*	0.19	*.661*	2.57	*.112*	2.27	*.132*	**4.49**	***.034***	**10.51**	***.002***

Means (SEM)	Acceptability	W1	*T*−1	*T*	*T* + 1	Wcrit	W6	W7	W8	LW
*All blocks*										
Definite—fulfilled	3.64 (0.04)	408 (2.40)	299 (1.67)	287 (1.43)	301 (1.89)	336(3.03)	331(2.22)	303 (1.75)	302 (1.84)	355 (3.53)
Definite-unfulfilled	1.58 (0.05)	399 (2.80)	299 (1.23)	290 (1,46)	309 (1.67)	345 (2.39)	342 (2.85)	304 (2.20)	297 (2.08)	336 (3.61)
Indefinite- fulfilled	3.55 (0.04)	399 (2.91)	302 (1.69)	293 (1.46)	312 (1.61)	340 (1.81)	330 (2.27)	300 (1.54)	299 (1.55)	339 (1.94)
Indefinite—unfulfilled	2.33 (0.02)	408 (2.23)	299 (1.26)	288 (1.12)	313 (1.89)	343 (2.10)	344 (2.05)	305 (1.46)	298 (1.93)	348 (3.11)

Factor	*F*	*p*	*F*	*p*	*F*	*p*	*F*	*p*	*F*	*p*	*F*	*p*	*F*	*p*	*F*	*p*	*F*	*p*	*F*	*p*
Determiner	**79.90**	** < *****.001***	0.01	*.919*	0.30	*.584*	1.16	*.282*	**6.76**	***.013***	0.07	*.793*	0.04	*.851*	0.01	*.943*	0.00	*.769*	0.27	*.609*
Fulfillment	**424.89**	** < *****.001***	0.00	*.956*	0.17	*.677*	0.09	*.769*	**4.00**	***.048***	4.00	*.051*	**17.18**	** < *****.001***	1.46	*.230*	1.19	*.283*	2.25	*.140*
Det x Ful	**133.52**	** < *****.001***	**7.77**	***.005***	0.38	*.536*	2.89	*.091*	1.17	*.194*	1.20	*.274*	0.17	*.684*	0.94	*.337*	0.64	*.429*	**16.69**	** < *****.001***

The results are depicted separately for all blocks (lower part) and the first block (upper part). Significant effects are highlighted by the bold type. Mean acceptability ratings and mean reading times (in ms) for each word and the standard error of the mean (SEM) computed according to a method proposed by Cousineau (2005) for within-participants designs are presented

**Table 2:** Overview over the results of Experiment 2

Means (SEM)	Acceptability	W1	W2	*T*−1	*T*	*T* + 1	*T* + 2	*T* + 3	LW
*First Block*									
Definite—fulfilled	3.46 (0.04)	480 (4.21)	372 (3.42)	350 (3.63)	346 (2.91)	342 (4.31)	382 (3.63)	404 (4.35)	419 (4.73)
Definite—unfulfilled	2.45 (0.56)	472 (4.43)	369 (2.80)	352 (3.29)	346 (2.99)	349 (3.47)	400 (4.17)	418 (4.66)	418 (3.85)
Indefinite—fulfilled	3.48 (0.04)	470 (4.41)	370 (2.88)	351 (2.96)	346 (3.24)	341 (4.16)	382 (3.57)	399 (4.02)	416 (4.54)
Indefinite—unfulfilled	2.58 (0.04)	466 (3.75)	365 (3.24)	344 (3.28)	344 (3.31)	351 (3.61)	396 (3.91)	421 (4.88)	443 (6.32)

Factor	*F*	*p*	*F*	*p*	*F*	*p*	*F*	*p*	*F*	*p*	*F*	*p*	*F*	*p*	*F*	*p*	*F*	*p*
Determiner	2.94	*.089*	2.45	*.127*	0.35	*.558*	0.43	*.518*	0.11	*.740*	0.00	*.947*	0.55	*.459*	0.02	*.901*	3.70	*.060*
Fulfillment	**127.65**	** < *****.001***	1.57	*.216*	1.20	*.274*	0.53	*.473*	0.14	*.713*	3.77	*.055*	**14.16**	** < *****.001***	**13.28**	** < *****.001***	**4.56**	***.038***
Det x Ful	1.13	*.290*	0.07	*.786*	0.00	*.949*	1.64	*.206*	0.07	*.788*	0.00	*.953*	0.59	*.442*	0.48	*.489*	**5.78**	***.020***

Means (SEM)	Acceptability	W1	W2	*T*−1	*T*	*T* + 1	*T* + 2	*T* + 3	LW
*All Blocks*									
Definite—fulfilled	3.58 (0.04)	410 (1.69)	324 (1.46)	308 (1.37)	310 (1.29)	309 (1.77)	328 (1.70)	337 (2.18)	349 (1.98)
Definite—unfulfilled	2.26 (0.05)	409 (1.68)	324 (1.04)	307 (1.24)	313 (1.49)	315 (1.50)	337 (1.64)	341 (2.12)	349 (2.10)
Indefinite—fulfilled	3.59 (0.04)	412 (1.50)	326 (1.48)	308 (1.05)	314 (1.25)	309 (2.11)	326 (1.60)	334 (1.63)	348 (1.55)
Indefinite—unfulfilled	2.35 (0.05)	408 (1.38)	322 (1.20)	306 (1.13)	315 (1.61)	318 (1.48)	332 (1.67)	343 (1.82)	357 (2.16)

Factor	*F*	*p*	*F*	*p*	*F*	*p*	*F*	*p*	*F*	*p*	*F*	*p*	*F*	*p*	*F*	*p*	*F*	*p*
Determiner	2.23	*.137*	0.01	*.904*	0.01	*.908*	0.09	*.767*	1.62	*.208*	0.54	*.467*	3.08	*.081*	0.08	*.779*	3.25	*.073*
Fulfillment	**191.28**	** < *****.001***	1.06	*.305*	0.77	*.384*	0.32	*.573*	1.32	*.251*	**9.91**	***.002***	**8.87**	***.004***	**5.79**	***.020***	2.18	*.145*
Det x Ful	1.76	*.186*	0.32	*.575*	1.40	*.239*	0.06	*.804*	0.19	*.666*	0.60	*.440*	0.18	*.671*	1.18	*.279*	**5.35**	***.022***

The results are depicted separately for all blocks (lower part) and the first block (upper part). Significant effects are highlighted by the bold type. Mean acceptability ratings and mean reading times (in ms) for each word and the standard error of the mean (SEM) computed according to a method proposed by Cousineau (2005) for within-participants designs are presented

**Table 3:** Overview over the results of Experiment 3

Means (SEM)	Acceptability	W1	W2	*T*−1	*T*	*T* + 1	*T* + 2	*T* + 3	Wcrit
*First Block*									
Which—singular	3.20 (0.04)	460 (5.23)	399 (3.48)	367 (3.50)	355 (3.09)	373 (5.41)	393 (5.32)	392 (4.08)	397 (4.57)
Which—plural	3.19 (0.04)	480 (5.55)	402 (3.79)	370 (3.15)	368 (5.55)	401 (5.70)	429 (5.31)	422 (4.95)	399 (3.56)
When—singular	3.31 (0.04)	481 (4.61)	407 (3.84)	367 (3.71)	358 (3.60)	382 (5.26)	405 (5.42)	401 (4.87)	389 (3.37)
When—plural	2.59 (0.05)	471 (5.82)	389 (4.11)	361 (3.45)	359 (3.56)	387 (5.24)	417 (5.39)	401 (4.36)	393 (3.98)

Factor	*F*	*p*	*F*	*p*	*F*	*p*	*F*	*p*	*F*	*p*	*F*	*p*	*F*	*p*	*F*	*p*	*F*	*p*
Sentence type	**11.00**	***.001***	0.74	*.395*	0.39	*.534*	1.78	*.183*	0.00	*.948*	0.12	*.729*	0.00	*.955*	0.94	*.340*	1.47	*.234*
Context	**38.99**	** < *****.001***	0.26	*.614*	2.11	*.147*	0.33	*.569*	1.55	*.213*	3.61	*.065*	**11.16**	***.002***	**5.12**	***.030***	0.73	*.397*
Type x Con	**48.15**	** < *****.001***	**4.05**	***.047***	**6.29**	***.012***	1.48	*.224*	1.29	*.256*	**4.97**	***.026***	**6.86**	***.009***	**6.94**	***.009***	0.03	*.862*

Means (SEM)	Acceptability	W1	W2	*T*−1	*T*	*T* + 1	*T* + 2	*T* + 3	Wcrit
*All Blocks*									
Which—singular	3.15 (0.04)	427 (2.64)	333 (1.38)	305 (1.19)	303 (1.02)	314 (3.78)	329 (2.84)	335 (2.58)	340 (2.91)
Which—plural	3.23 (0.04)	437 (2.60)	338 (1.58)	308 (1.21)	306 (1.45)	332 (3.76)	351 (2.49)	348 (3.28)	343 (1.75)
When—singular	3.42 (0.04)	433 (2.55)	337 (1.56)	306 (1.19)	303 (1.25)	314 (4.05)	331 (3.09)	332 (2.76)	335 (3.35)
When—plural	2.37 (0.05)	432 (2.56)	334 (1.68)	307 (1.17)	305 (1.36)	327 (3.98)	348 (3.47)	343 (2.16)	347 (2.24)

Factor	*F*	*p*	*F*	*p*	*F*	*p*	*F*	*p*	*F*	*p*	*F*	*p*	*F*	*p*	*F*	*p*	*F*	*p*
Sentence type	**22.86**	** < *****.001***	0.00	*.984*	0.00	*.962*	0.08	*.776*	0.07	*.796*	1.06	*.309*	0.04	*.849*	2.56	*.118*	0.05	*.816*
Context	**65.44**	** < *****.001***	2.06	*.160*	0.21	*.647*	1.14	*.293*	1.86	*.173*	**4.23**	***.044***	**12.68**	** < *****.001***	**6.73**	***.012***	**5.86**	***.017***
Type x Con	**103.66**	** < *****.001***	2.77	*.096*	**5.10**	***.024***	0.77	*.381*	0.04	*.832*	1.28	*.258*	1.39	*.238*	0.29	*.587*	1.90	*.170*

Means (SEM)	W9	W10	W11	LW
*First Block (continued)*				
Which—singular	379 (4.50)	399 (5.47)	425 (5.21)	432 (5.70)
Which—plural	376 (3.23)	403 (4.22)	418 (5.13)	425 (5.63)
When—singular	386 (3.73)	409 (4.37)	427 (5.24)	446 (5.92)
When—plural	386 (3.73)	416 (3.72)	421 (4.35)	432 (5.12)

Factor	*F*	*p*	*F*	*p*	*F*	*p*	*F*	*p*
Sentence type	2.10	*.156*	1.33	*.252*	0.01	*.913*	1.36	*.250*
Context	0.43	*.512*	1.57	*.215*	0.48	*.491*	1.63	*.210*
Type x Con	0.01	*.933*	0.04	*.851*	0.00	*.974*	0.33	*.566*

Means (SEM)	W9	W10	W11	LW
*All Blocks (continued)*				
Which—singular	333 (3.70)	344 (3.35)	364 (3.80)	375 (3.25)
Which—plural	329 (2.51)	339 (2.72)	357 (2.72)	363 (2.76)
When—singular	330 (2.12)	342 (2.23)	355 (2.39)	369 (2.36)
When—plural	336 (1.73)	348 (1.84)	356 (1.94)	373 (2.75)

Factor	*F*	*p*	*F*	*p*	*F*	*p*	*F*	*p*
Sentence type	0.33	*.566*	0.50	*.480*	0.78	*.378*	0.11	*.738*
Con	0.22	*.639*	0.05	*.818*	0.63	*.430*	1.04	*.310*
Type x Con	3.14	*.078*	2.74	*.102*	1.23	*.270*	**5.02**	***.027***

The results are depicted separately for all blocks (lower part) and the first block (upper part). Significant effects are highlighted by the bold type. Mean acceptability ratings and mean reading times (in ms) for each word and the standard error of the mean (SEM) computed according to a method proposed by Cousineau (2005) for within-participants designs are presented

**Table 4:** Overview over the results of Experiment 4

Means (SEM)	Acceptability	W1	W2	W3	W4	*T*−1	*T*	*T* + 1	*T* + 2
*First block*									
Active—fulfilled	3.37 (0.05)	470 (5.71)	391 (3.50)	377 (4.32)	385 (4.36)	378 (4.21)	372 (3.78)	388 (4.48)	375 (3.35)
Active—unfulfilled	2.22 (0.04)	479 (5.62)	385 (3.82)	378 (3.79)	375 (3.71)	367 (3.97)	363 (4.06)	383 (4.13)	373 (3.74)
Passive—fulfilled	3.57 (0.05)	488 (5.53)	386 (3.97)	372 (3.95)	378 (4.68)	367 (3.98)	365 (3.30)	389 (4.34)	379 (3.09)
Passive—unfulfilled	2.43 (0.06)	486 (5.29)	386 (3.34)	368 (3.24)	384 (3.29)	376 (4.10)	364 (2.83)	391 (4.73)	378 (3.81)

Factor	*F*	*p*	*F*	*p*	*F*	*p*	*F*	*p*	*F*	*p*	*F*	*p*	*F*	*p*	*F*	*p*	*F*	*p*
Sentence type	**13.33**	** < *****.001***	3.51	*.070*	0.09	*.759*	1.60	*.214*	0.07	*.789*	0.12	*.735*	0.78	*.377*	1.13	*.289*	0.48	*.452*
Fulfillment	**186.46**	** < *****.001***	0.72	*.399*	0.64	*.428*	0.00	*.964*	0.05	*.823*	0.04	*.842*	0.67	*.415*	0.16	*.685*	0.05	*.816*
Type x Ful	0.28	*.602*	0.55	*.458*	0.11	*.742*	1.20	*.274*	1.40	*.238*	2.55	*.115*	0.28	*.595*	0.07	*.793*	0.37	*.541*

Means (SEM)	Acceptability	W1	W2	W3	W4	*T*−1	*T*	*T* + 1	*T* + 2
*All Blocks*									
Active—fulfilled	3.44 (0.05)	438 (2.79)	332 (1.98)	311 (1.66)	316 (1.76)	316 (1.98)	314 (1.79)	326 (1.51)	330 (1.53)
Active—unfulfilled	2.02 (0.04)	438 (2.52)	330 (1.79)	311 (1.61)	312 (1.92)	315 (1.80)	310 (1.74)	323 (1.85)	326 (1.89)
Passive—fulfilled	3.63 (0.04)	447 (2.23)	328 (1.43)	310 (1.47)	316 (1.55)	315 (1.55)	312 (1.26)	327 (1.44)	330 (1.43)
Passive—unfulfilled	2.16 (0.05)	441 (2.70)	329 (1.59)	309 (1.17)	312 (1.10)	316 (1.31)	312 (1.43)	326 (1.66)	327 (1.45)

Factor	*F*	*p*	*F*	*p*	*F*	*p*	*F*	*p*	*F*	*p*	*F*	*p*	*F*	*p*	*F*	*p*	*F*	*p*
Sentence type	**13.14**	** < *****.001***	2.37	*.132*	1.48	*.224*	0.33	*.566*	0.02	*.896*	0.01	*.922*	0.01	*.916*	0.48	*.492*	0.00	*.950*
Fulfillment	**365.91**	** < *****.001***	0.71	*.405*	0.02	*.900*	0.03	*.870*	2.27	*.136*	0.02	*.899*	0.29	*.589*	0.47	*.492*	2.10	*.158*
Type x Ful	1.38	*.248*	0.73	*.394*	0.13	*.715*	0.16	*.687*	0.04	*.850*	0.11	*.740*	0.88	*.351*	0.03	*.869*	0.03	*.863*

Means (SEM)	*T* + 3	Wcrit	W11	LW
*First block (continued)*				
Active—fulfilled	400 (3.91)	476 (7.74)	438 (5.03)	439 (6.00)
Active—unfulfilled	393 (3.59)	514 (8.57)	477 (7.11)	446 (5.79)
Passive—fulfilled	394 (2.65)	438 (8.04)	406 (5.23)	428 (5.14)
Passive—unfulfilled	408 (4.13)	511 (6.99)	458 (6.81)	453 (7.07)

Factor	*F*	*p*	*F*	*p*	*F*	*p*	*F*	*p*
Sentence type	0.61	*.439*	**6.78**	***.011***	**12.58**	** < *****.001***	0.59	*.444*
Fulfillment	1.31	*.256*	**25.33**	** < *****.001***	**49.58**	** < *****.001***	**8.23**	***.006***
Type x Ful	**4.39**	***.036***	**4.84**	***.028***	0.22	*.643*	0.59	*.443*

Means (SEM)	*T* + 3	Wcrit	W11	LW
*All blocks (continued)*				
Active—fulfilled	355 (1.62)	445 (5.62)	385 (2.80)	373 (2.06)
Active—unfulfilled	348 (1.91)	474 (5.90)	408 (3.76)	374 (2.49)
Passive—fulfilled	352 (1.81)	426 (6.22)	367 (3.10)	368 (2.68)
Passive—unfulfilled	355 (2.04)	481 (6.17)	401 (3.50)	379 (2.74)

Factor	*F*	*p*	*F*	*p*	*F*	*p*	*F*	*p*
Sentence type	0.35	*.556*	1.57	*.217*	**6.48**	***.014***	0.05	*.817*
Fulfillment	0.21	*.649*	**16.69**	** < *****.001***	**49.89**	** < *****.001***	2.80	*.099*
Type × Ful	**4.64**	***.034***	**8.22**	***.006***	1.97	*.167*	2.89	*.092*

The results are depicted separately for all blocks (lower part) and the first block (upper part). Significant effects are highlighted by the bold type. Mean acceptability ratings and mean reading times (in ms) for each word and the standard error of the mean (SEM) computed according to a method proposed by Cousineau (2005) for within-participants designs are presented

## Footnotes

1 The presentation of all possible context-test-sentence pairs for each participant may be seen as somewhat unusual in the context of psycholinguistic studies. In the context of experimental psychological studies, however, this procedure is preferred in order to ensure a good signal-to-noise ratio through a sufficient number of repetitions. However, in order to assess whether the organization and presentation of the sentence material in sentence sets might have influenced the results by, for example, strategic considerations or by memory for previous presentations, we carried out two analyses, one main analysis including all blocks and an additional analysis in which we only analyzed trials in the first block. Because of our block organization, this latter analysis included only one example sentence per set, so potential processing strategies or memory effects should not play a role. The results of the main analyses and the additional analysis are summarized (for all experiments) in tables in the appendix. It becomes clear that the results of the two analyses do not differ significantly from one another and actually show a very high degree of overlap. For this reason and because we prefer to present and discuss results based on data with a higher signal-to-noise ratio, we focus our discussion on the main analysis (analysis of all blocks).

2 Since longer RTs can be significant under certain circumstances, we performed all analyses with the longer RT included. These analyses did not produce significantly different results for any of the three experiments.
